# Use of mental health supports by civilians exposed to the November 2015 terrorist attacks in Paris

**DOI:** 10.1186/s12913-020-05785-3

**Published:** 2020-10-20

**Authors:** Philippe Pirard, Thierry Baubet, Yvon Motreff, Gabrielle Rabet, Maude Marillier, Stéphanie Vandentorren, Cécile Vuillermoz, Lise Eilin Stene, Antoine Messiah

**Affiliations:** 1 Non Communicable Diseases and Trauma Division, Santé Publique France, French National Public Health Agency, F-94415 Saint-Maurice, France; 2 Team MOODS, CESP, Inserm, Université Paris-Saclay, UVSQ, 94807 Villejuif, France; 3 CESP, Inserm, Université Sorbonne Paris Nord, Villetaneuse, France; 4 APHP, Hôpital Avicenne, Bobigny, France; 5 Centre National de Ressources et de Résilience (CN2R), Lille/Paris, France; 6 Department of Social Epidemiology, INSERM, Sorbonne Université, Institut Pierre Louis d’Epidémiologie et de Santé Publique, F75012 Paris, France; 7 Support, Data Treatment and Analysis Division, Santé Publique France, French National Public Health Agency, Saint-Maurice, France; 8 Scientific and International Division, Santé Publique France (The French Public Health Agency), Saint-Maurice, France; 9 INSERM, Bordeaux Population Health Research Center, UMR 1219, Univ Bordeaux, F-33000 Bordeaux, France; 10 Norwegian Centre for Violence and Traumatic Stress Studies (NKVTS), Oslo, Norway

**Keywords:** Terrorist attacks, Mental health services, Post-traumatic, Stress disorders, Health service research, Disaster medicine, Psychological first aid, Mental health outreach, Health care use

## Abstract

**Background:**

The use of mental health supports by populations exposed to terrorist attacks is rarely studied despite their need for psychotrauma care. This article focuses on civilians exposed to the November 2015 terrorist attacks in Paris and describes the different combinations of mental health supports (MHSu) used in the following year according to type of exposure and type of mental health disorder (MHD).

**Methods:**

*Santé publique France* conducted a web-based survey of civilians 8–11 months after their exposure to the November 2015 terrorist attacks in Paris. All 454 respondents met criterion A of the DSM-5 definition of post-traumatic stress disorder (PTSD). MHD (anxiety, depression, PTSD) were assessed using the PCL-5 checklist and the Hospital Anxiety and Depression Scale. MHSu provided were grouped under outreach psychological support, visits for psychological difficulties to a victims’ or victim support association, consultation with a general practitioner (GP), consultation with a psychiatrist or psychologist (specialist), and initiation of regular mental health treatment (RMHT). Chi-squared tests highlighted differences in MHSu use according to type of exposure (directly threatened, witnessed, indirectly exposed) and MHD. Phi coefficients and joint tabulations were employed to analyse combinations of MHSu use.

**Results:**

Two-thirds of respondents used MHSu in the months following the attacks. Visits to a specialist and RMHT were more frequent than visits to a GP (respectively, 39, 33, 17%). These were the three MHSu most frequently used among people with PTSD (46,46,23%), with depression (52,39,20%), or with both (56,58, 33%). Witnesses with PTSD were more likely not to have RMHT than those directly threatened (respectively, 65,35%). Outreach support (35%) and visiting an association (16%) were both associated with RMHT (Phi = 0.20 and 0.38, respectively). Very few (1%) respondents initiated RMHT directly. Those who indirectly initiated it (32%) had taken one or more intermediate steps. Visiting a specialist, not a GP, was the most frequent of these steps.

**Conclusion:**

Our results highlight possibilities for greater coordination of mental health care after exposure to terrorist attacks including involving GP for screening and referral, and associations to promote targeted RMHT. They also indicate that greater efforts should be made to follow witnesses.

## Background

Terrorist attacks take a heavy psychosocial toll on the lives of people who have been directly threatened, witnesses, and those indirectly exposed (i.e., who learn that a loved one has been threatened, injured or killed). This is reflected in a high prevalence of acute stress and mental health disorders (MHD) – specifically, post-traumatic stress disorder (PTSD), major depressive disorder and anxiety-based disorders - in the months, and even years, following the attack [1–4]. These MHD negatively impact families, social relationships and work capacity. They can also induce or aggravate other morbidities such as addictive disorders, suicidal ideation and somatic disorders [5, 6]. It is therefore important to meet the needs of people suffering from MHD in a timely fashion, and to provide appropriate treatment to reduce the intensity and duration of these consequences and associated social complications. Some studies recommend providing early and proactive outreach psychological support to satisfy the particular treatment needs of populations exposed to terrorist attacks [7, 8]. Collective expertise recommends acute stress disorder management in the first month after exposure [9–11]. Experts consider that after the first month, effective interventions for PTSD need to include regular mental health treatment (RMHT) (for example 8 to 12 sessions of trauma-focused cognitive behavioural therapies) [12]. Other morbidities often found after psychotraumatic exposure, for example depression, require and often benefit from effective treatments such as antidepressant drugs and cognitive behavioural therapies [13]. It is also important to allocate enough resources to meet the increased, and often long-term, demand for mental health supports (MHSu) after disasters [14, 15]. The persistent problem of insufficient access to mental health care for those who need it [16] is all the more salient in the context of care after a terrorist attack. Access to MHSu is particularly important for people directly affected by a collective massive attack [17–19], as they represent the exposure group with the highest risk of psychological sequelae [18]. Yet this access may differ depending on whether the person was directly threatened, was a witness during the attack, or learned that a loved one was threatened, injured or killed.

On 13 November 2015, 3 bomb attacks were perpetrated near a football stadium in Saint-Denis, in the northern suburbs of Paris. That same day, three shootings and 1 bombing also occurred in restaurants in central Paris, as did the slaughter and taking hostage of civilians during a rock concert at the Bataclan theatre, also located in central Paris. The attacks killed 130 people, injured 643 [20], and led to serious psychotraumatic exposure for several thousands of people. On 18 November 2015, local residents were witnesses to an assault by the police in Saint-Denis on the terrorists who took part in the 13 November attacks.

Emergency outreach psychosocial support units (French abbreviation: CUMP) were immediately deployed following the attacks [7, 20]. There are 100 CUMP throughout the country, each comprising trained psychiatrists, psychologists and nurses. Their mission is to deliver primary psychosocial support to people who have been exposed (directly threatened, witness, loved one of victim), to identify these people’s needs for immediate care and provide specific emergency psychological care, to refer them to a psychiatrist and/or a psychologist (‘specialist’ hereafter) if necessary, and to raise awareness in those exposed of the event’s potential psychological consequences [20–22]. Following the Paris attacks, several CUMP immediately provided support to those who asked for it in the streets, and later in ad hoc information and reception centres which were set up in the town halls of the affected districts in central Paris and in Saint-Denis, as well as in the National Military School, Institute of Forensic Medicine and Hôtel-Dieu hospital, all located in central Paris. All these centres were dismantled after 1 month. France’s peacetime public and private health care system was responsible for providing longer-term psychological care. The French Ministry for Health subsequently sent a document to all those officially registered as victims by the Ministry of Justice, providing them with free access to healthcare, including consultations with a specialist [23, 24]. Furthermore, people who considered themselves victims of terrorism could also contact one of the existing permanent victims’ support associations managed by the Ministry of Justice, free of charge. These associations provide welcome centres, social listening services, psychological support, social and legal assistance, and contact information to connect with local healthcare partners [25]. The people concerned were informed about these victims’ support associations by CUMP team members, police officers, peers, etc. Finally, several non-governmental associations, created either by victims of the November 2015 attacks or victims of previous traumatic events, offered support.

Most post-terrorist attack studies examine the psychosocial impact on those exposed. However, the literature on mental healthcare use after a mass traumatic event is scarce [26]. More specifically, few studies address the use of different types of MHSu [14, 15, 27]. Furthermore, virtually nothing is known about combined MHSu use, despite the fact that specific combinations of MHSu may have specific consequences on public health (e.g., in terms of optimization and appropriateness of care provided). Analysis of correlations between different types of MHSu and their joint-tabulation may help identify these related patterns and associated public health issues. Further research on the use of MHUs is essential therefore to improve the psychological care of people at risk. Any such research should take into account the specificities of each country’s health system and cultural aspects [28].

ESPA 13 November (*Enquête de Santé Publique post-Attentats du 13 Novembre*) is a web-based survey which launched on 7 July 2016 and ended on 10 November 2016. Developed by *Santé publique France* (the French national public health agency), it aimed to document the psychological effects of the November 2015 attacks and the use of crisis-related and peacetime healthcare services by civilians and first responders directly threatened, witnesses, and those indirectly exposed. The analysis of the impact of the attacks on first responders using data from ESPA 13 November has been treated in other articles [29]. For the present study, we used the survey’s data to make a detailed investigation of exposed civilians’ self-declared use of different types of MHSu. Our specific objectives were to:

- estimate the use of different types of MHSu according to the type of exposure to the attacks and the probable related MHD;
- analyse the combinations of different types of MHSu and their correlations, and in turn identify patterns of mental health support use;

in order to provide information to guide future policies for the care of populations exposed to mass traumatic events such as terrorist attacks.

## Methods

### Study population and data collection

Civilians participating in the ESPA 13 November survey were over 15 years of age, had been exposed to the 13 November terrorist attacks or the 18 November police assault, and met criterion A of the Diagnostic and Statistical Manual of Mental Disorders (DSM–5) definition of PTSD [30] in at least one of the following ways: direct exposure (i.e., directly threatened) (A1), witnessing the trauma directly (A2), and learning that a loved one had been exposed (i.e., death or injury) to the trauma (A3).

*Santé publique France* used radio, television, and press advertisements to contact persons eligible for the survey. Furthermore, collaboration with key stakeholders (victims’ and victim support associations) to relay information to citizens started 2 months before the survey launched, and continued throughout the survey period. Moreover, coordinators from some CUMP agreed to send an information letter about the survey to exposed persons who they had provided emergency care to, and whose contact addresses they had kept. In addition, a door-to-door survey information campaign was carried out in collaboration with restaurants and cafés affected by the attacks and/or police assault, and letters were dropped into the mailboxes of households within a radius of 100 m of each affected area in August and September 2016.

Civilians interested in participating were invited to complete an online (civilian-specific) inclusion questionnaire on the *Santé publique France* website to determine their eligibility and to provide informed consent. Eligible participants were then directed to a web-based epidemiological questionnaire on the same site. For the inclusion questionnaire, 856 separate internet connections were recorded. Of these, 222 persons were not eligible and 5 were eligible but refused to participate. Of the remaining 629 eligible candidates who agreed to participate, 53 completed the inclusion questionnaire twice. Accordingly, 576 civilians were directed to the epidemiological questionnaire. Of these, 526 provided sufficient information to allow exposure classification, and 454 answered questions about their use of MHSu.

### Study variables

The ESPA 13 November survey questionnaire was designed between March and June 2016 in a context of relative urgency. Questions were formulated by field-based actors and victims’ associations working in close partnership in order to ensure that relevant information - both for the field-based actors and from a public health point of view- would be collected. The construction of the questionnaire was also based on the IMPACT study [3] questionnaire, in order to guarantee that future pooled analyses could be carried out. Wherever possible we used existing and scientifically validated measurement tools. A scientific board was set up to advise on the choice of tools to use, and to validate the content of the questionnaire.

#### Socio-demographic characteristics

As MHD and the choice of MHSu used by people may be associated with demographics and socioeconomic characteristics, the following information was collected in the questionnaire: gender, age, educational level (*no high school diploma, high school diploma, Bachelor’s degree or Post-graduate degree*), socio-professional category (*craft worker/trader/business leader, executive/professor/senior intellectual, employee/blue-collar worker, intermediate profession (*e.g.*, technician), no professional activity, other*), professional situation (*professionally active, student, retired, unemployed, full-time home maker),* matrimonial status (*married, in a civil union or common-law relationship, single, divorced, widowed)* (Table 1).

**Table 1:** Demographics, exposure type, and self-reported MHSu use, ESPA_13_November Survey, *N* = 454

Age (mean)	N	Mean
	454	Mean = 40 Standard Deviation: 12.4
	N	%
**Gender**	452	
Female	299	66%
Male	153	34%
**Educational level**	454	
No high school diploma	36	8%
High school diploma	45	10%
Bachelor’s degree or higher	373	82%
**Socio-professional category**	450	
Craft worker, trader, business leader	28	6%
Executive, professor, senior intellectual	250	56%
Employee, blue-collar worker	67	15%
Intermediate profession (e.g., technician)	31	7%
No professional activity	24	5%
Other	50	11%
**Professional situation**	453	
Professionally active	355	78%
Student	39	9%
Retired	26	6%
Unemployed	27	6%
Full-time home maker	6	1%
**Matrimonial situation**	454	
Married, in a civil union, or common-law relationship	246	54%
Single	176	39%
Divorced	27	6%
Widowed	5	1%
**Exposure**	454	
Directly threatened	158	35%
Witness	208	46%
Indirectly exposed	88	19%
**PTSD**	438	
No	276	63%
Yes	162	37%
**Probable anxiety disorder**	451	
No	153	34%
Yes	298	66%
**Probable depressive disorder**	450	
No	310	69%
Yes	140	31%

#### Exposure

For each of the different attack/police assault locations, a set of questions corresponding to the specific scenario (Additional files 1 and 2) was asked in order to ensure that the various elements of the participant’s exposure met the criteria for defining PTSD in the DSM 5, i.e. people were exposed to death, the threat of death, actual or threatened serious injury, or actual or threatened sexual violence, in one of the following ways:

- Direct exposure: physically injured, hit by a bomb blast, targeted or shot at by the terrorists inside the Bataclan theatre, or targeted or shot at either in the outside seating area or in the main room of the restaurants attacked);
- Witnessing the attacks: whether by sight, sound, smell or touch;
- Indirect exposure by learning that a relative or close friend was exposed to a trauma (learned that a loved one had been killed, injured or threatened).

#### Psychological state

Psychological state was explored using two validated psychometric scales:

- The PCL-5 checklist to assess probable PTSD (Cronbach alpha coefficient = 0.93).
  This 20-item checklist assesses the presence of the 20 trauma-related symptoms defined in 6 (A to F) of the 8 criteria (A to H) of the DSM-5 for PTSD. The 6 criteria are as follows: A “exposure to traumatic event” (explained above), B “intrusion”, C “persistent avoidance of trauma-related stimuli”, D “negative cognitions/mood”, E “alterations in arousal”, F “symptoms from criteria A-E last for at least one month”. For the present study, in order to assess probable PTSD, we also inserted a question to assess criterion G (“distress/interference with different areas of life”) of the DSM-5. Specifically, participants had to report whether their symptoms caused them difficulties in at least one of the following 4 aspects of everyday life: relationships with family, with friends, at work, globally. People defined with probable PTSD (i.e., meeting criteria A to G) were regarded as needing treatment [31];
- the “Hospital Anxiety and Depression scale” (HAD) (14 items with scores from 0 to 3, Cronbach alpha coefficient = 0.87) [32]. A score greater than or equal to 8 on either the anxiety (HAD-a) or depression (HAD-d) subscales identifies, respectively, the presence of probable anxiety and depressive disorders warranting clinical assessment and management [32, 33].

#### Mental-health support use

Participants were asked whether they had received any MHSu since the events using various questions. These questions were developed with the active participation of the healthcare stakeholders involved during and after the attacks/assault. They took into account the experiences of victims and the specificities of the French healthcare system. When developing the questions, the stakeholders involved took into account the fact that a large proportion of the respondents would most probably not have a clear recollection when exactly they had used a MHSu. Greater emphasis was therefore placed in the questions concerning the different stages of care sought after the attacks (immediate support, outreach reception centres, peacetime public and private health care systems). More specifically, respondents were first asked if they remembered receiving support immediately after the attacks using the following questions: “*Do you have any memories of what you experienced between the immediate aftermath of the event and the moment when you returned to your living accommodation?” (“Yes”/ “No”) If “Yes” “Do you remember receiving any support or assistance?” If “Yes” “Who provided this support or assistance?” (Multiple choice answer: Firefighters, SAMU (*i.e.*, organisations providing emergency medical services), CUMP, Non-identified personnel, Other healthcare providers”); Can you tell us in which place(s)? (Multiple choice answer: At the scene of the attack, In the street, Hôtel-Dieu, Other hospital, Paris city district town hall, Police office, Elsewhere, I do not know)*. Second, they were asked if they had received psychological support during a visit to one of the ad hoc information and reception centres set up in the days immediately following after the attacks: “*After the events (OR “after returning to your living accommodation” for people on or close to the scene at the time of the attacks/assault), did you go to any of the reception centres which were set up in the days following the attacks? (Multiple choice answer: Paris city 10th district town hall or adjacent Parmentier school, 11th district town hall, Saint-Denis town hall, Institute of Forensic Medicine, National Military School, Hôtel-Dieu hospital, Other” (“yes” / “No”). If “Yes”, “Did you receive psychosocial support from health professionals there (CUMP, army health services, etc.)?”.* Third, participants were asked whether aside from the ad hoc care provided, they had seen or consulted a healthcare provider for psychological problems since the terrorist attacks: “*Since the events, aside from the places mentioned above, have you seen or been consulted by a person(s) from a public organization, an association or a private practice for your psychological problems?” (“Yes” / “No). If “yes”, “What public organisation, association or private practice was (were) the person(s) a part of?”* Multiple choice answer: “*Hospital emergency services, Specialized hospital consultant for psychotrauma, Medical psychological centre (CMP), CUMP, Specialized private consultant, an Association in the “FRANCE VICTIMES” federation (*e.g.*, ‘Paris aid to victims’, ADAVIP 92), a Victims’ association (*e.g.*, AFVT –FENVAC, 13onze15, Life for Paris), the French medico-social children’s association Ose, a General practitioner, I don’t know, and other”*. Participants were also asked whether they had been hospitalized for psychological difficulties since the terrorist attacks or hospitalized for physical injuries, and whether they had received psychological support from a specialist (i.e., psychiatrist or psychologist) during hospitalization. Although the specific type of therapy followed and regular treatment initiation date were also questionnaire items, we assumed that some participants would have difficulty remembering this information. Accordingly, for the analyses, we only used answers to the primary question about RMHT initiation: “*Since the events, have you initiated regular psychological care?*”

MHSu were classified into the following 5 categories: outreach psychological support (OPS), visits for psychological difficulties to a victims’ association or a victim support association, consulting a general practitioner (GP), consulting a specialist, and initiation of RMHT.

Participants were classified as having received OPS if (Table 2):

- medico-psychological professionals had provided them support immediately after the event either in the street or close to the place of the attacks, or if they had received psychological support during a visit to one of the ad hoc information and reception centres during the first month following the attacks.
- support was given in the month following the attacks by a CUMP member, a psychologist at the police station, a psychologist or occupational physician as part of a specific occupational medicine support system set up on an emergency basis by their employer;

**Table 2:** Answers to questionnaire items focusing on use of mental health supports, ESPA_13_November *N* = 454

Type of mental health support (MHSu)	Number (%)
**Outreach psychological support (OPS)**	159 (35)
Psychiatrist or psychologist in the street immediately after event	29
Occupational psychologist or physician	7
Police psychologist	14
OPS in ad hoc information and field reception centre	
Hôtel-Dieu hospital	35
Institute of Forensic Medicine	8
National Military school	15
Town hall in affected district (Paris, St-Denis)	83
Emergency psychosocial support unit (CUMP)	29
**Consultation with a specialist (psychiatrist or psychologist)**	178 (39)
*Specialized hospital consultant for psychotrauma*	47
Specialized private consultant	92
Hospital emergency service	15
*Medical Psychological Centre* (CMP)	36
Hospitalised for psychological problems	11
Hospitalisation for physical injury	22
**Contact with a member of an association**	**71 (16)**
Victims’ support association(FRANCE VICTIMES federation)	44
Victims’ association	35
**Medical visit to a GP’s office (GP)**	**79 (17)**
**Regular mental health treatment (RMHT)**	**151 (33)**
**Combinations of different MHSu**	**Numbers (%)**
**Consultation with a specialist or RMHT**	454
No	223 (49)
Yes	231 (51)
**Use of at least one MHSu**	454
**No**	148 (33)
**Yes**	306 (67)

Persons provided psychological support by a member of a victims’ association or victim support association were classified as having received psychological support from an association.

Consulting a GP was defined as a medical visit to a GP’s office.

Persons provided psychological support by a specialist in a hospital emergency unit, in a dedicated psycho-trauma unit, or during hospitalization, and those who consulted a specialist in a public psychological consultation centre were classified as having consulted a specialist (Table 2).

Persons who answered “yes” to the question “*Since the events, have you initiated regular psychological care?*” were classified as having initiated RMHT.

More details about the questions asked and the classification of participants’ responses can be found in Additional files 1, 2 and 3.

### Statistical analyses

The proportions of the different MHSu used were assessed overall, and both according to type of exposure (being threatened, witnessing, indirectly exposed) and MHD (anxiety, depression, probable PTSD).

For each MHSu, Chi-2 tests were used to test the independence of the distributions between the use or not of the given MHSu, exposure groups and MHD. To investigate patterns of MHSu use, we first computed Phi coefficients to measure associations between the different MHSu. Since MHSu variables were measured dichotomously (i.e., “Yes/No”), Phi is equivalent to the usual correlation coefficient [34]. Second, we explored the different combinations between MHSu by joint tabulation of the 5 types of supports. Analyses were performed using SAS Enterprise Guide 7.11. Phi confidence intervals were estimated with the bootstrap technique using R version 3.5.1 [35].

### Ethics

When answering the web-based epidemiological questionnaire, respondents had access to free telephone support (from 10 am to 10pm, Monday to Saturday) provided by specially trained psychologists. The ESPA 13 November survey was approved by the *Commission Nationale de l’Informatique et des Libertés* (CNIL) (authorization demand n°915262v2, deliberation n°2016–209 of 7 July 2016) and the Committee for the Protection of Persons (amendment number 7035/3/3283). All those who visited the *Santé publique France* website, whether or not they finally participated in the survey, had access to information about the possible consequences of exposure to these attacks and about how they could seek care. Participants under 18 years of age had to provide signed authorization from their parents.

## Results

### Respondent characteristics and mental health support use

Most respondents were women (66%), middle aged (mean 40 years), professionally active (78%) and highly educated (82% Bachelor’s degree or higher) (Table 1). With regard to exposure, 35% had been directly threatened, 46% were witnesses and 19% were indirectly exposed (70 had lost a loved one and 18 were close to someone injured or directly threatened).

Thirty-seven percent of the study sample had probable PTSD. Prevalence rates of probable depressive (HAD-d > 7) or anxiety (HAD-a > 7) disorders were 31 and 66%, respectively. Twenty-three percent of the sample had probable anxiety disorder but not PTSD.

Overall, 35% of the study sample reported receiving OPS. With regard to the other types of MHSu, 17% had consulted their GP, 16% had met a person from an association providing support for psychological problems, and 39% had consulted a specialist in a structure belonging to the peacetime health care system (Table 2). One third had initiated RMHT. Overall therefore, 67% of respondents had used at least one MHSu, and 51% had either consulted a specialist or initiated RMHT.

### Use of mental health supports according to type of exposure

**Fig. 1:**
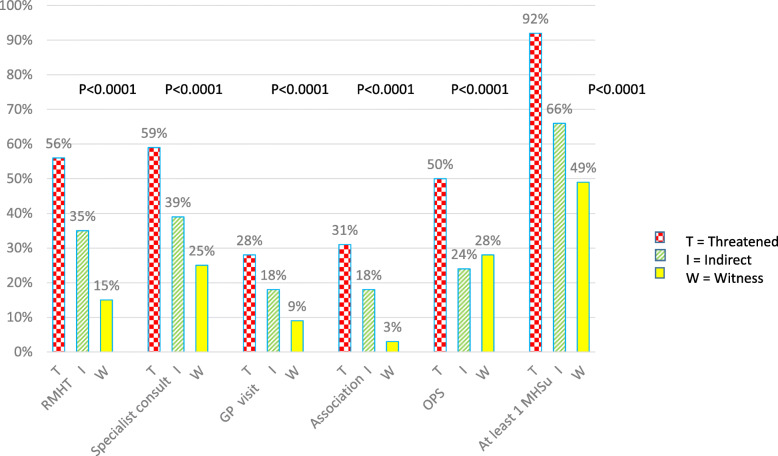
Proportions of users by exposure for each MHSu (Chi-2_p), ESPA_13_ November, *N* = 454

Civilians directly threatened were twice as likely as witnesses or persons indirectly exposed to have received OPS (50% vs. 24 and 28%, respectively) (*p* < 0.001) (Fig. 1). An increasing gradient (*p* < 0.001), depending on exposure category, was observed for the other 4 MHSu as follows: witness (3% associations, 9% GP consultations, 25% specialist consultations, 15% RMHT), indirectly exposed (18,18,39,35%), and directly threatened (31,28,59,56%).

**Fig. 2:**
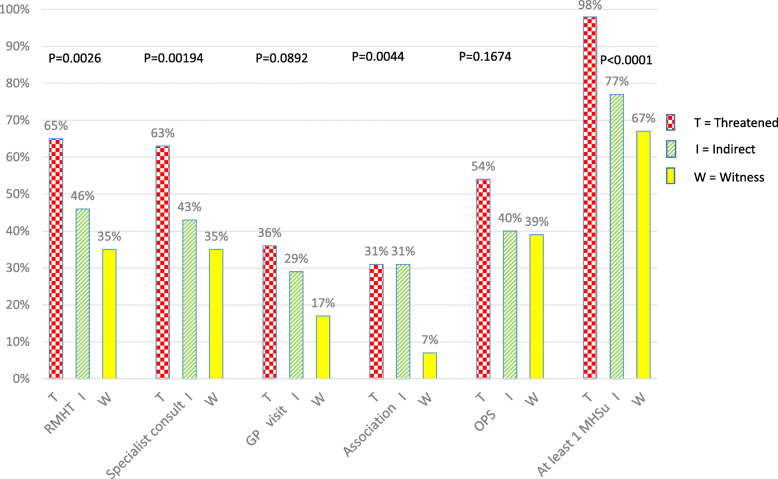
Proportions of users by exposure for each MHSu (Chi-2_ p) for respondents with PTSD, ESPA_13_ November, *N* = 162

Proportions of OPS use were high in people with probable PTSD, and were not significantly different between witnesses, those indirectly exposed and those directly threatened (39% vs. 40 and 54%) (Fig. 2). Witnesses (7% associations, 35% specialist consultations, 35% RMHT) less frequently reported going to an association (*p* = 0.004), consulting a specialist (*p* = 0.002) and initiating RMHT (*p* = 0.003) than those indirectly exposed (31, 43, 46%) and those directly threatened (31, 63, 65%).

### Use of different MHSu according to type of probable mental health disorder

**Fig. 3:**
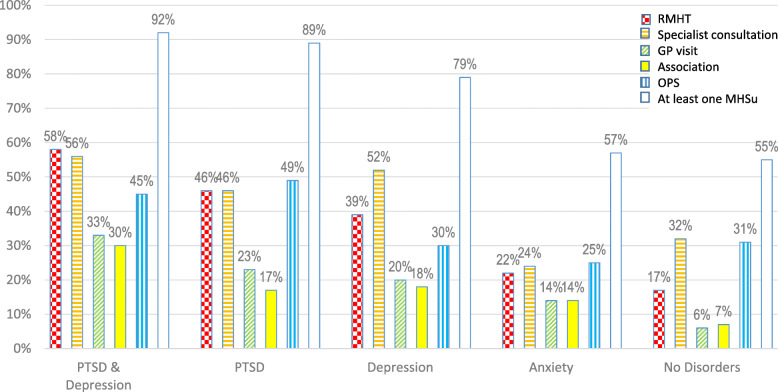
Proportions of each MHSu use according to probable mental health disorder, ESPA_13_November, *N* = 454

All MHSu were used by participants in all groups classified according to the type of probable MHD (Fig. 3). Over half of those with anxiety only, or with no disorder whatsoever had used a support (57 and 55%, respectively). This value rose to 79% among those with depression only, 89% among those with probable PTSD only, and 92% among those with both PTSD and depression.

The proportion of participants who had consulted a specialist was higher among people with PTSD (46%), those with depression (52%), and those with both (56%), than among participants with anxiety only (24%) and those with no disorder (32%) (*p*-value < 0.0001). For all groups classified according to the type of probable MHD consulting a specialist was the most frequent or second most frequent MHSu used.

An increasing gradient was observed in the proportions of RMHT, from those without any disorder to those with anxiety only, to those with depression only, to those with PTSD only, and finally to the most symptomatic group, which had both probable PTSD and depression (17 to 58%, *p* < 0.0001). This was also the case for visits to GP (6 to 33%, *p* < 0.0001). The use of OPS was higher among people with PTSD (45 to 49%) than those without PTSD (25 to 31%), (*p* = 0.0035). However, even among the latter, between a quarter and a third used OPS, depending on the probable MHD.

Meeting with an association was much more frequent for persons with both PTSD and depression than for others (30% vs 7 to 18%), *p* = 0.0004).

### Patterns of MHSu use

We analysed correlations and combinations to highlight associations (or the lack thereof) between the different uses of MHSu in order to identify related patterns.

### Correlations

MHSu were significantly correlated with each other in terms of use, except for contacting an association and consulting a specialist (Table 3). RMHT was strongly correlated with consulting a specialist (Phi = 0.38) and with contacting an association for psychological problems (Phi = 0.38) (Table 3). The correlations between GP consultations and other MHSu varied from 0.17 (contacting an association) to 0.28 (RMHT). For OPS, the correlation with other MHSu was 0.20 for initiating RMHT and consulting a GP, 0.10 for consulting a specialist, and 0.10 for contacting an association for psychological problems.

**Table 3:** *p*-values of Chi-2 test, Phi coefficients between MHSu, 95% Confidence Intervals ESPA_13_November, *N* = 454

Type of MHSu	Outreach psychological support	Victims’ or Victim support Association	Visit to General practitioner	Consultation with a psychologist or psychiatrist	Regular Mental health treatment
		P of Chisq PHI [CI95%]	P of Chisq PHI [CI95%]	P of Chisq PHI [CI95%]	P of Chisq PHI [CI95%]
Outreach psychological support	1	*P* = 0.0276 0.10 [0.01–0.20]	*p* < 10^−4^ 0.20 [0.10–0.29]	*P* = 0.0376 0.10 [0.01–0.19]	*p* < 10^− 4^ 0.20 [0.10–0.29]
Victims’ or Victim support Association		1	*P* = 0.0003 0.17 [0.06–0.28]	*P* = 0.9986 0.00 [−0.10–0.02]	*p* < 10^− 4^ 0.38 [0.29–0.47]
Visit to General practitioner			1	*p* < 10^− 4^ 0.20 [0.11–0.30]	*p* < 10^− 4^ 0.28 [0.19–0.37]
Consultation with a psychologist or psychiatrist				1	*p* < 10^− 4^ 0.38 [0.29–0.47]
Regular mental health treatment					1

### Mental health support combinations

The concatenation of the 5 types of MHSu resulted in 30 combinations of MHSu use (Table 4), unevenly distributed. Four main patterns stood out and concerned 60% of the participants: those who did not use any MHSu (*N* = 148, 33%), those who reported only OPS (*N* = 49, 11%), those who consulted a specialist but did not engage in RMHT (*N* = 41, 9%), and those who consulted a specialist and had RMHT (*N* = 33, 7%).

**Table 4:** Combinations of uses of the 5 types of MHSu (OPS, associations, GP, specialist, RMHT) ESPA_ 13_ November

OPS	Association	GP	Psychiatrist or psychologist	Regular mental health treatment	Number (%)
N	N	N	N	N	148 (33)
Y	N	N	N	N	49 (11)
N	N	N	Y	N	41 (9)
N	N	N	Y	Y	33 (7)
Y	N	N	Y	Y	21 (5)
Y	N	N	Y	N	20 (4)
N	Y	N	N	Y	13 (3)
Y	N	Y	Y	Y	12 (3)
N	N	Y	Y	Y	10 (2)
N	Y	N	N	N	9 (2)
Y	N	N	N	Y	9 (2)
N	Y	N	Y	Y	8 (2)
N	N	Y	N	N	8 (2)
N	N	Y	Y	N	8 (2)
Y	Y	N	N	Y	8 (2)
Y	Y	Y	Y	Y	8 (2)
Y	Y	Y	N	Y	7 (1)
N	N	N	N	Y	6 (1)
Y	N	Y	Y	N	6 (1)
Y	N	Y	N	N	5 (1)
Y	Y	N	Y	Y	4 (1)
	N	Y	N	Y	4 (1)
N	Y	Y	Y	Y	3 (1)
N	N	Y	N	Y	3 (1)
N	Y	N	Y	N	2 (< 1)
N	Y	Y	N	Y	2 (< 1)
Y	Y	N	N	N	2 (< 1)
Y	Y	N	Y	N	2 (< 1)
Y	Y	Y	N	N	2 (< 1)
N	Y	Y	Y	N	1 (< 1)

*N* No, *Y* Yes

Sixty-seven percent of the sample used at least one MHSu. One percent directly accessed RMHT (i.e., with no declared intermediate steps), for example GP or specialist consultations, while 32% accessed it after one or more intermediate steps.

Thirty-five percent reported receiving OPS. Of these, 11% reported receiving only OPS, 10% had also visited their GP, and 10% had consulted a specialist without going through a GP. Only 2% of those who received OPS but did not contact an association or consult a GP or specialist, subsequently initiated RMHT.

In total, 17% of all participants had visited a GP. Of these, 3% reported that they had not visited a specialist or initiated RMHT, 4% had initiated RMHT without first consulting a specialist, and 11% reported having consulted a specialist.

Thirty-nine percent of the study participants reported consulting a specialist within the pre-existing (i.e. peacetime) healthcare system. Of these, 42% (i.e., 19% of the whole study sample) had neither received OPS nor visited a GP, while 54% (i.e., 21%) had initiated RMHT.

Sixteen percent of the study sample had contacted a victims’ association or victim support association for their psychological problems. Just under half of these (7%) had also received OPS. Three-quarters (12%) of them had also initiated RMHT. Only 2% of the study sample had only contacted an association.

## Discussion

Comparing our rates of MHSu use with those measured after other terrorist attacks is difficult because health care systems vary from country to country. For example, France has dedicated psycho-social support units which provide emergency psychological support to persons involved in mass traumatic events. In addition, victim profiles, the amount of time elapsed between an attack and the launch of an investigative study, as well as the tools used to measure MHSu use, may all differ between studies and countries. In our study, 67% of the participants had used at least a MHSu since the November 2015 attacks in Paris. More specifically, 51% had either consulted a specialist or started RMHT. In contrast, Boscarino et al. estimated that approximately 45% of New York City residents with associated PTSD or severe depression within 1 year of the 11 September 2001 attacks in the USA, had received psychological counselling from a professional (psychiatrist, doctor, counsellor, victims’ association, etc.) [17]. Another study conducted 6 months after these attacks on a sample of New York City residents showed that 15% of those directly affected and 36% of those with PTSD or depression had visited a healthcare professional or association [18]. A third study, conducted 3 to 6 months after September 11 among Manhattan residents showed that only 27% of people with severe symptoms of PTSD or depression were being treated [36].

A study following people directly threatened in the Oklahoma city bombing (19 April 1995) reported similar results to ours, with 69% of the exposed civilian population having received some kind of mental health intervention during the first 6 months after the bombing (debriefing, mental health professional, GP, pastor) [37].

Few studies have been performed in Europe on post-disaster MHSu use. A survey on the people exposed to the attacks on Utøya Island in 2011 showed that 69% used a specialised mental health service between 5 and 15 months after the event [14] and were provided care within the framework of a proactive prevention outreach programme. This programme systematically assigned a local primary care contact person who was responsible for organizing 3 mental health screenings during the year after the terrorist attacks and who, if necessary, referred an exposed person to an appropriate service.

The similarities and differences observed between all the above-mentioned studies may – at least in part - be explained by the different nature and modalities of exposure. In our study, the intensity and duration of exposure may have played a role in the relatively high use of MHSu. The survivors on Utøya island and in the Bataclan theatre were exposed for more than one hour to an immediate and direct threat on their lives, and witnessed a massacre with no possible escape. Instead, most of the US studies cited above concerned residents as well as those directly exposed. One correlate of exposure is the high psychological impact (for example 37% of participants in our study had probable PTSD) which in turn encourages the use of MHSu. Furthermore, data from the European Study of the Epidemiology of Mental Disorders (ESEMeD) suggest that in normal circumstances (i.e., outside of massive traumatic events), access to MHSu appears to be more frequent in Europe than in the USA or Canada [38]. Resource availability can also play a role. In the ESEMeD study, France ranked first among 6 countries (Belgium, France, Italy, Netherlands, Spain, UK) in terms of the availability of GP (161 per 100,000 citizens) and psychiatrists (22 per 100,000 citizens) in 2001 [39]. Levels of healthcare insurance and out-of-pocket expenditures may also contribute to differences in the use of MHSu [39]. In the US studies cited above, two of the factors associated with MHSu use were having a GP [17, 18] and having health insurance [19, 40, 41]. The French health care system provides universal coverage and a designated referring GP for each insured person. Furthermore, in the case of the November 2015 attacks, the fact that the French government provided persons recognized as victims with free healthcare services - in the form of visits to GP and specialists - probably encouraged the use of MHSu. On the other side socio-cultural factors, such as a lack of knowledge and negative beliefs about mental disorders, can negatively impact MHSu use, leading to underutilisation [16, 39]. Finally, we cannot exclude selection bias, given that some of our participants were contacted by CUMP psychiatrists.

Half of those directly threatened in our study had received OPS, this figure dropping to approximately a quarter for witnesses and those indirectly exposed. The level of OPS use might be based on both the quantity of immediate OPS deployed by authorities after a massive traumatic event and the manifestation of the need for MHSu by the persons exposed. In our study, OPS use was significantly higher among persons who we classified as having probable PTSD. However for this group, no significant difference was seen in terms of exposure type. This result suggests that the OPS deployed immediately after the November 2015 attacks met a need for MHSu in people for whom exposure significantly impacted their subsequent mental health. Post-disaster studies highlight the importance of urgently implementing MHSu systems that help meet the exceptional - both immediate [7, 27] and longer term [8, 14, 27] - needs of a disrupted community [40]. These systems seem to facilitate the subsequent recovery of people who are mentally injured [7, 8]. In our study, OPS use was also positively associated with RMHT. One hypothesis to explain this is that outreach systems raise exposed persons’ awareness of the value of contacting a specialist should they experience psychological symptoms at a later date.

Only 17% of persons exposed to terrorist attacks in our study (28% among those directly threatened) reported visiting their GP for psychological problems. This result contradicts the general preconceived belief that the GP is the primary or first healthcare provider who people turn to when they have psychological problems [8, 40]. It also runs counter to results from the ESEMeD study, where GP were reported to be the main healthcare professionals by all participants in France who had used MHSu for psychological problems at some point in their lifetime (GP lifetime consultation rate of 73% in France) [39]. Furthermore, in our study, the majority of those who had seen a GP had also either consulted a specialist or initiated RMHT. This may be a result of patients’ own initiative, but may also suggest that the GP concerned preferred to first screen and then refer patients to a specialist rather than treat them alone.

The most frequently reported MHSu was consulting a specialist (39%). Interestingly, 72% of all those who consulted a specialist did not see a GP. This high proportion might be partly due to greater post-attack accessibility to specialists, especially as specialist density per capita is higher in Paris than in the rest of France [42–44]. It may also be partly due to the fact that the French government provided free healthcare, in the form of visits to specialists, in the wake of the attacks [23, 24].

Contacting a victims’ association or a victim support association was not correlated with consulting a specialist. However, it was correlated with RMHT. Victim support associations and most victims’ associations employ psychologists. It is therefore possible that participants who contacted these associations did not need to consult a specialist in a private office or in a hospital. In addition, representatives of the associations may have encouraged the patient to initiate RMHT.

Among the MHD examined, probable PTSD and probable depression were the main determinants for consulting specialists, visiting GP and initiating RMHT. Persons with probable concomitant PTSD and depression used MHSu more than others. This higher rate of MHSu use in people suffering from depressive disorder or co-morbidities than the rate of use observed in those who suffered from anxiety disorders, has been observed in numerous studies outside the context of terrorist attacks [38, 39, 45, 46]. Indeed, experts recommend taking into account comorbidities - such as depression, toxicological addictions, and suicidal risk - when treating PTSD, as they are very common [47].

In a posttraumatic context, such as that following a terrorist attack, recommendations highlight the importance of active monitoring and mental health screening of persons at risk of PTSD, and of offering individual trauma-focused cognitive behavioural therapy to adults with clinically serious symptoms of PTSD [12]. However, in our study, a substantial proportion of people with probable PTSD (35% of those directly threatened, 65% of witnesses, and 54% of those indirectly exposed) did not initiate RMHT. The non-use of MHSu by people with mental disorders is common, both in persons not exposed to any collective traumatic event [16] and in exposed persons in the aftermath of an attack [28].

A special effort must be made to promote care for witnesses to attacks. Indeed, in our study, witnesses with probable PTSD consulted specialists less frequently than those directly threatened. Furthermore, the proportion of participants with PTSD reporting that they had not used any MHSu was higher for witnesses than for persons directly threatened.

Finally, all MHSu investigated here were used to some extent in our study sample. Specifically, 67% of the sample used at least one MHSu. This suggests that the variety of care supports offered responded to the diversity of needs of the exposed population following the attacks. Outreach MHSu met the immediate and real MHSu needs of those exposed. Contacting a victims’ association possibly lightened the workload for specialists in public and private healthcare structures, and offered an alternative to people who may have feared they would be stigmatised. GP may have acted as a first contact point who then referred patients to a suitable specialist. Unfortunately, the ESPA 13 November questionnaire only asked for the date of RMHT initiation, not for the dates of visits to GP or specialists. It was therefore not possible to assess the degree to which the ‘standard’ accepted cascade of care (i.e., use of outreach psychological support, referral by the GP or a specialist, and initiation of RMHT) was followed.

### Limitations

Our results should be interpreted with the limitations of the survey methodology in mind. The participant selection method certainly introduced selection bias. It is possible that at the time of the survey, those suffering the most from the November 2015 attacks in Paris and Saint Denis may have felt that it was too difficult or too early to participate. This would lead to an underestimation of treatment needs [40]. In the Utøya cohort study, those who did not participate in the first wave were more likely to have more post-traumatic stress reactions during the second wave of the study than participants in the first wave [48]. This finding was not echoed in a French open cohort study which followed people directly threatened in the January 2015 terrorist attacks in Paris [49]. It is also possible that people who were suffering less at the time of the ESPA 13 November survey may have felt less motivated to participate or indeed that their participation was less legitimate than that of others.

As the survey was web based, exposed people with no internet access were excluded. Accordingly, those most socially disadvantaged were probably underrepresented [40]. Furthermore, the vectors used to convey information about the survey (general meetings of victims’ associations, letters from certain emergency psychiatrists) probably contributed to the high observed rates of use of MHSu.

It was legally not possible to have a list of the victims with which to compare our list of participants, a strategy recommended by Schlenger et al. [50]. The number of people who technically could have been eligible for our survey may reach several thousands. Indeed, as of March 2018, 1685 victims were on the Ministry of Justice’s list, while a total of 2650 people were eligible for financial compensation because they had been wounded or were directly threatened, were witnesses, or had been indirectly exposed through the death of a close relative [51]. According to the Ministry of Justice, no exhaustive victims list exists. It is therefore not possible to calculate precisely the participation rate or to compare non-participants with participants, or indeed to calibrate the sample.

As the ESPA 13 November survey was web-based, there was no clinical examination, despite this being the reference diagnostic method. Moreover, self-assessment may have resulted in inaccuracy in individual diagnosis. Furthermore, the interview period lasted 4 months, and encompassed the date of the subsequent terrorist attack in Nice (14 July 2015) [52]. This attack may have led to an increase in the intensity of PTSD symptoms in our sample. According to a meta-analysis, PTSD may not decrease in intensity during the first year in populations directly threatened in intentional traumatic events [53]. Even if this is true, the intensity of psychological symptoms and, consequently, the level of use of MHSu for someone who participated in the ESPA 13 November survey just after its launch in July 2016 (i.e., directly after the Nice attack), may have been higher than they would have been had the same person joined the study later on.

As data on MHSu care consumption in our study was only collected from participants’ self-reports, that is to say not independently collected, our results are prone to recall biases. Some studies suggest that self-reported data overestimate care consumption, but this overestimation concerns more the number of visits for the same MHSu than the number of different MHSU available [40, 54]. Finally, the cross-sectional nature of the study prevented us from assessing whether the observed correlations might be causal.

### Strengths

Our study explored both the use of MHSu existing independently of collective events (i.e., GP visits, mental health specialist consultations) and MHSu in the form of outreach care and associations, by civilian victims of a terrorist attack who were either directly threatened, were witnesses to the attack or had a close family member or friend directly threatened, injured or killed. Our study results highlight that treatment coverage was better for those directly threatened than for witnesses or indirectly exposed people. This result supports evidence from the IMPACT study which was also carried out after the January 2015 Paris terrorist attacks, and which showed differences in the levels of utilisation of immediate medico-psychological care according to exposure type (i.e., witness vs directly threatened) for civilians during the first 48 h after the events [3]. Indeed, these complementary results underline a public health issue in the French care system: despite the need for exposed persons to receive mental health treatment, the level of access to different MHSu currently depends on the type of exposure.

The second main study strength is that the involvement of various stakeholders ensured that its design would be more relevant in terms of the issues to study (for example by addressing MHSu provided by associations and by ad hoc outreach psychological services).

Third, this survey has, to our knowledge, the highest number of civilian participants of all French studies to date addressing the impact of terrorist attacks on civilian adults who meet criterion A of the DSM-5 definition for PTSD [3, 4, 55, 56], and this fact allowed us to analyse combinations of MHSu and related patterns.

Fourth, the use of web-based questionnaires is known to facilitate responses to intimate questions, the latter being more difficult to answer in face-to-face interviews [50]. Furthermore, we used validated scales for screening the main mental disorders examined, with good sensitivity and specificity [31, 33, 57, 58]. Although these scales measure the intensity of symptoms, they may not sufficiently assess the need for care [59]. This is why, in addition to DSM-5 criteria A,B,C,D, and E, we also considered the negative impact of PTSD symptoms on everyday life (criterion G “functional significance”).

## Conclusions

This study, based on data from the ESPA 13 November survey, provides important results for policy-making, as it highlights the serious psychological impact of the November 2015 terrorist attacks in Paris on exposed civilians and offers a picture of the latter’s use of a comprehensive panel of available MHSu. Most users of the field-based immediate outreach psychological services, which were set up in the aftermath of the event, also visited a GP and/or a specialist in the 8 to 11 months following the attacks. This stresses the necessity to coordinate all these MHSu, not only in emergency situations, but also in the medium term, in order to strengthen the effectiveness of psycho-trauma management. Provided that the ethical rules for the protection and security of personal data are respected, systems to monitor the mental health of exposed people as well as their use of MHSu should be put in place. GP are a useful resource in this context, and should be at least sensitized and trained in screening and referral to appropriate management services for mental disorders following terrorist attacks. Our results also highlight the beneficial role of victims’ associations and victim support associations as key actors in the MHSu network, in addition to the social and legal assistance they provide. Furthermore, our findings indicate that greater efforts should be made to identify and provide care to witnesses to terrorist attacks and individuals indirectly exposed, not just those directly threatened. Finally, people presenting post-trauma comorbidities are the biggest users of MHSu, stressing the importance of establishing strategies to treat these situations.

ESPA 13 November is an ongoing open cohort study. A second wave of web-based questionnaires is planned for 2020. This will be complemented by the collection of retrospective and prospective data on participants’ health care consumption (i.e., not only MHSu), based on health insurance data records (e.g., visits to private GP or specialists, prescriptions reimbursed by France’s universal health care system). Both data sets should provide us with a more complete perspective of the evolution of MHSu use and general care consumption by civilians since their exposure to the terrorist attacks in November 2015 in Paris and Saint Denis.

## Supplementary information

**Additional file 1.** Relevant sections used in the civilian version of the French web-based questionnaire for Phase 1 of the ESPA_ 13_ November study. Additional file 1 is a French language clean-copy of the relevant sections of the web-interview guide used as part of the present study’s design (these sections dealt with the person’s current social and demographic situation; the ways in which the person was exposed to aggression, physical injuries, the loss or exposure of a loved one, the psychological consequences of this exposure, and the different aspects of related psychological care).

**Additional file 2.** Translation of the relevant variables used in the civilian version of the French web-based questionnaire for Phase 1 of the ESPA_ 13_November study. Additional file 2 is the English language clean-copy version of the questions from the web-interview guide used in the construction of the variables analysed for this article.

**Additional file 3.** Questions and criteria used to collect civilians’ self-reported information on use of mental-health supports (MHSu) for the ESPA_ 13_ November study. Additional file 3 contains two tables. Table A presents the questions used to collect self-reported information about the use of mental-health supports, table B presents the criteria used to distribute the answers to the different variable items according to the different types of MHSu.

## Data Availability

The datasets generated and/or analysed during the current study are not publicly available due to its submission to GDPR obligations (the datasets is composed of personal data) but are available from the corresponding author on reasonable request (request which must be compliant with GDPR obligations).

## Abbreviations

ANR: French National Research Agency
CNIL: C*ommission Nationale de l’Informatique et des Libertés*
CMP: *Medical-Psychological Centre*
DSM-5: Diagnostic and Statistical Manual of Mental Disorders, fifth edition
ESPA 13 November: *Enquête de Santé Publique post-Attentats du 13 Novembre*
CUMP: Emergency psycho-social support unit
GP: General practitioner
HAD: Hospital Anxiety and Depression scale, anxiety subscale (HAD-a) or depression subscale (HAD-d)
MHSu: Mental-health support
MHD: Mental health disorder
OPS: Outreach psychological support
PCL-5: DSM-5 checklist for PTSD
PTSD: Post-traumatic stress disorder
RMHT: Regular mental health treatment
