# Supplementary material for: Use of mental health supports by civilians exposed to the November 2015 terrorist attacks in Paris
Source: BMC Health Serv Res. 2020 Oct 20;20:959. doi: 10.1186/s12913-020-05785-3 (PMC7574168; doi:10.1186/s12913-020-05785-3)
Supplement: Supplementary file 1 — Additional file 1. Relevant sections used in the civilian version of the French web-based questionnaire for Phase 1 of the ESPA_ 13_ November study. Additional file 1 is a French language clean-copy of the relevant sections of the web-interview guide used as part of the present study’s design (these sections dealt with the person’s current social and demographic situation; the ways in which the person was exposed to aggression, physical injuries, the loss or exposure of a loved one, the psychological consequences of this exposure, and the different aspects of related psychological care). [file 12913_2020_5785_MOESM1_ESM.pdf]

Additional file 1: Relevant sections used in the civilian version of the French web-based questionnaire for Phase 1 of the ESPA_ 13_ November study

xxxx: topics of the original questionnaire containing the questions used for the article: "Use of mental-health supports by civilians exposed to the November 2015 Terrorist Attacks in Paris"

Xxxxxx: topics of the original questionnaire not used and not presented in this excerpt

QUESTIONNAIRE IN FRENCH

Bienvenue sur la page d'accueil du questionnaire [2]« non intervenants (population civile) » de l'enquête ESPA 13 novembre.

Afin de naviguer dans le questionnaire, veuillez ne pas utiliser le bouton « retour en arrière » de votre navigateur.
Vous pouvez revenir à cette page d'accueil en cliquant sur le logo Santé publique France.
Pour vous rendre sur les différentes parties vous pouvez :

- Cliquer sur chacune des parties dans le menu à gauche
- Cliquer sur le titre de la partie de votre choix sur cette page d'accueil
- Cliquer sur le bouton « sauvegarder et changer de page » lorsque vous vous arrivez à la fin d'une partie.

Vous trouverez ci-dessous une brève description des thèmes qui seront abordés.

Votre situation actuelle

Ces données sont classiquement recueillies dans les enquêtes épidémiologiques. Ce sont des grands facteurs qui permettent le regroupement des résultats en fonction de différentes catégories et qui peuvent influer sur votre état de santé ou votre recours aux soins : âge, sexe, situation professionnelle, niveau d'étude.

Votre expérience personnelle des événements

Ces questions vont vous permettre d'exprimer objectivement et subjectivement ce que vous avez vécu et de quelle(s) façon(s) vous avez été impliqué(e) dans ces attentats.

Deux échelles psychométriques (Questionnaire des expériences de dissociation péritraumatique [PDEQ] et partie A3 du questionnaire Shortness of breath, tremulousness, racing heart and sweating rating scale [STRS]) vont également évaluer vos réactions émotionnelles aigues. Ces réactions peuvent influer sur votre état psychologique.

Vos blessures physiques et leurs conséquences

Ces questions vont vous permettre d'exprimer les difficultés rencontrées du fait de vos blessures physiques. Il est important de pouvoir les prendre en compte pour construire une prise en charge globale associant une réparation somatique, psychique et sociale des personnes blessées physiquement et psychotraumatisées.

Concernant vos proches

Cette partie aborde différents aspects que vous avez eu à affronter lors de la perte d'un proche dans ces attentats : annonce du décès, recherche de votre ou vos proches, constats du décès. Ces informations sont utiles pour aider à un meilleur accompagnement des personnes endeuillées.

Votre vécu des conséquences de l'événement

Le vécu des conséquences des attentats et leurs répercussions dans la vie personnelle et professionnelle sont indispensables pour identifier des situations aggravantes de retentissement du traumatisme ainsi que pour aider à une prise en charge adaptée qui associe à l'aide psychologique un soutien social pertinent. Cette partie aborde également certains problèmes de santé autres que psychologiques qui peuvent apparaître ou s'aggraver suite à des évènements traumatisants ainsi que la consommation de substances psychoactives (drogues, alcool, tabac etc.).

Vos antécédents traumatiques et psychologiques

Cette courte partie explore l'existence d'antécédents traumatiques et psychologiques notables, au cours de votre vie avant les événements de novembre 2015, ainsi que le vécu de situations difficiles l'année précédente, en 2014.

Ce que vous éprouvez psychologiquement

Quatre questionnaires explorant les retentissements psychologiques pouvant se développer chez certaines personnes exposées à des événements traumatisants vont vous être posés. Ces questionnaires abordent l'état de stress post-traumatique, les symptômes anxieux et dépressifs, le risque suicidaire et le deuil compliqué (si vous avez perdu un proche lors des attentats). L'analyse des réponses à ces questionnaires permettra d'évaluer l'adéquation entre les symptômes et les soins psychiques qui ont pu être reçus et ainsi de proposer des pistes d'amélioration de la prise en charge.

Le soutien psychologique que vous avez reçu

Cette partie aborde le soutien et les soins psychologiques que vous avez pu recevoir dans les suites immédiates des attentats jusqu'à aujourd'hui. Ces informations sont indispensables afin de permettre d'améliorer la prise en charge proposée au décours d'événements traumatiques.

Votre entourage

Il s'agira d'explorer les soutiens dont vous pouvez bénéficier autour de vous. C'est un des facteurs associés à l'évolution des psycho-traumatismes.

Votre vécu du questionnaire

Ces courtes questions nous permettront d'avoir un retour sur la façon dont vous avez vécu la passation de ce questionnaire.

Vos remarques

Trois questions ouvertes portant sur les enfants, le changement et les associations de victimes vous sont proposées. Un espace libre vous est également offert pour exprimer ce que vous souhaitez.

S'il vous est difficile de remplir le questionnaire du fait de blessures physiques, de difficultés avec le français ou avec l'outil informatique, ou si vous vous sentez mal en remplissant le questionnaire, vous pouvez appeler ce numéro afin de prendre contact avec un psychologue (du lundi au samedi de 10h à 22h) : **N° non surtaxé - Info Conseil : 09 70 14 99 60**

Votre situation actuelle

Sexe

- [ ] Homme
- [ ] Femme

Année de naissance (aaaa)

Situation matrimoniale

- [ ] Marié(e), pacsé(e) ou en union libre
- [ ] Célibataire
- [ ] Divorcé(e)
- [x] Veuf / Veuve

Situation professionnelle

- [x] En activité professionnelle
- [ ] Au chômage
- [ ] Au foyer
- [ ] Étudiant
- [ ] Retraité

Selon vous, de quelle catégorie votre profession se rapproche-t-elle le plus ?

- [ ] Agriculteurs exploitants
- [ ] Artisans, commerçants, chefs d’entreprise
- [ ] Employés, ouvriers
- [ ] Sans activité professionnelle
- [ ] Cadres, prof. intellectuelles sup.
- [ ] Professions intermédiaires (technicien)
- [x] Autre

Niveau d'étude

- [ ] Certificat d'études primaires (6 ans)
- [ ] Formation professionnelle (CAP, BEP...)
- [x] Brevet des collèges
- [ ] Baccalauréat
- [ ] Études supérieures

○ Pas de diplôme

Votre expérience personnelle des événements

Dans quel(s) événement(s) avez-vous été impliqué(e) (vous étiez sur le lieu même, vous étiez proche géographiquement et vous vous êtes senti menacé) ?

Stade de France

- ○ Oui
- ◉ Non

Rues Bichat et Alibert : Le Petit Cambodge, Bar le Carillon

- ○ Oui
- ◉ Non

Rue de la Fontaine au Roi : La Casa Nostra et La Bonne Bière

- ○ Oui
- ◉ Non

Rue de Charonne : La Belle Equipe

- ○ Oui
- ◉ Non

Boulevard Voltaire : Le Comptoir Voltaire

- ○ Oui
- ◉ Non

Le Bataclan

- ○ Oui
- ◉ Non

Assaut du 18 novembre à Saint-Denis

- ○ Oui
- ◉ Non

Stade de France

Lorsque les explosions se sont produites, vous étiez

(•) À l'intérieur du stade de France

( ) À l'extérieur du stade de France

Avez-vous pensé que vous ou l'un de vos proches courriez un grave danger immédiat ?

( ) Oui

( ) Non

Pouvez-vous nous expliquer en quoi vous vous êtes senti menacé(e) ?

Stade de France

Lorsque les explosions se sont produites, vous étiez

- ( ) À l'intérieur du stade de France
- (•) À l'extérieur du stade de France

Où étiez-vous lorsque les terroristes ont déclenché leur ceinture d'explosifs ?

- ( ) A moins de 20 mètres des terroristes
- ( ) Entre 20 et 50 m des terroristes
- ( ) Plus loin et vous avez vu les explosions et/ou leur conséquences

	Oui	Non
	( )	( )
**Avez-vous vu l'explosion ? [explosion]**	( )	( )
**Avez-vous senti l'odeur de la poudre ou une autre odeur particulière provenant des lieux de l'événement ? [odeur_poudre]**	( )	( )
**Avez-vous ressenti le souffle de l'explosion ? [souffle_explo]**	( )	( )
**Avez-vous vu du sang ? [sang]**	(•)	( )
**Avez-vous vu des personnes inertes, blessées, mutilées, mortes ou des restes de corps humains [vu_personnes]**	( )	( )
**Avez-vous touché des personnes blessées ou mortes ? [touchee_blesses]**	( )	( )
**Avez-vous été blessé(e) ? [blesse]**	( )	( )
**Avez-vous reçu des projections de sang ou de restes de corps humains ? [projection]**	( )	( )
**Avez-vous eu peur d'être blessé ? [peur_blesser]**	( )	( )
**Avez-vous eu peur de mourir ? [peur_mourir]**	( )	( )
**Avez-vous pu apporter de l'aide ? [apporter_aide]**	( )	( )

	Oui	Non
	○	○
**Avez-vous éprouvé un sentiment ou eu une perception de la situation non listés ici ? [sentiments]**	◉	○

Préciser ce sentiment ou perception :

[ ]

Rues Bichat et Alibert : Le Petit Cambodge, Bar le Carillon ; Rue de la Fontaine au Roi : La Casa Nostra et La Bonne Bière ; Rue de Charonne : La Belle Equipe

Où étiez-vous lorsque les terroristes ont ouvert le feu ?

○ Dans le café visé ou à sa terrasse

○ Dans la rue à proximité des cafés visés

○ Dans une rue adjacente

	Oui	Non
	○	○
**Avez-vous fui l'événement pour vous mettre à l'abri ? [champ_jx13]**	○	○
**Avez-vous senti l'odeur de la poudre ou une autre odeur particulière provenant des lieux de l'événement ? [champ_hqst]**	○	○
**Avez-vous vu du sang ? [champ_uz3v]**	○	○
**Avez-vous vu les terroristes ? [champ_n29i]**	○	○
**Avez-vous croisé le regard des terroristes ? [champ_ah99]**	◉	○
**Vous êtes-vous senti visé(e) par les tirs ? [champ_u42a]**	○	○
**Avez-vous vu directement une personne être menacée, blessée ou mourir ? [champ_r89o]**	○	○
**Avez-vous vu des personnes inertes, blessées, mutilées, mortes ? [champ_vqi3]**	○	○
**Avez-vous touché des personnes blessées mutilées ou mortes ? [champ_rekf]**	○	○
**Avez-vous cru que vous alliez mourir ? [champ_d4i4]**	◉	○

	Oui	Non
	○	○
**Avez-vous été blessé(e) ? [champ_zh31]**	◉	○
**Vous êtes vous retrouvé(e) dans l’impossibilité d’apporter de l’aide ? [champ_o28p]**	○	○
**Avez-vous éprouvé un sentiment ou eu une perception de la situation non listés ici ? [champ_phgv]**	◉	○
**Avez-vous pu aussi apporter de l’aide ? [apporter_aide2]**	◉	○

Préciser ce sentiment / perception

Boulevard Voltaire

Où étiez-vous lorsque le terroriste a déclenché sa ceinture d’explosifs ?

( ) Dans le café visé ou à sa terrasse

( ) Dans la rue à proximité des cafés visés

( ) Dans une rue adjacente

	**Oui**	**Non**
	( )	( )
**Avez-vous vu le terroriste ? [champ_imk6]**	( )	( )
**Avez-vous vu l’explosion ? [champ_s42s]**	( )	( )
**Avez-vous senti l’odeur de la poudre ou une autre odeur particulière provenant des lieux de l’événement [champ_w8x6]**	( )	( )
**Avez-vous ressenti le souffle de l’explosion ? [champ_er27]**	(•)	( )
**Avez-vous vu du sang ? [champ_j6wo]**	( )	( )
**Avez-vous vu des personnes inertes, blessées, mutilées, mortes ou des restes de corps humains ? [champ_royz]**	( )	( )
**Avez-vous touché des personnes blessées, mutilées ou mortes ? [champ_wo5d]**	( )	( )
**Avez-vous reçu des projections de sang ou de restes de corps humains ? [champ_k48p]**	(•)	( )
**Avez-vous été blessé(e) ? [champ_i943]**	(•)	( )
**Avez-vous cru que vous alliez mourir ? [champ_ua0s]**	(•)	( )
**Avez-vous pu aussi apporter de l’aide ? [champ_m4u8]**	(•)	( )
**Avez-vous éprouvé un sentiment ou eu une perception de la situation non listés ici ? [champ_o6ub]**	(•)	( )

Préciser ce sentiment / perception

Bataclan

Vous étiez :

- (•) A l'intérieur du Bataclan
- ( ) A proximité immédiate du bataclan

Avez-vous pu vous enfuir à l'extérieur du Bataclan ?

- ( ) Oui
- ( ) Non

Etait-ce :

- ( ) Par le toit
- ( ) Par une issue de secours
- ( ) Autre

Etiez-vous dans la fosse ?

- ( ) Oui
- ( ) Non

Avez-vous pu vous cacher à l'intérieur du Bataclan ?

- ( ) Oui
- ( ) Non

Etait-ce :

- ( ) Dans un faux plafond
- ( ) Dans les toilettes
- ( ) Dans un local technique
- ( ) Dans la loge
- ( ) Dans les bureaux
- ( ) Dans une boutique de tatouage
- ( ) Sous le bar
- ( ) Dans les coulisses
- ( ) Autre

	Oui	Non
	○	○
Avez-vous senti l'odeur de la poudre ou une autre odeur particulière provenant des lieux de l'événement ? [champ_rj29]	○	○
Avez-vous vu du sang ? [champ_pd12]	○	○
Avez-vous vu des personnes inertes, blessées, mutilées ou mortes ? [champ_qw51]	○	○
Avez-vous touché des personnes blessées ou mortes ? [champ_ughd]	○	○
Avez-vous vu directement quelqu'un être menacé, blessé ou mourir ? [champ_zfgq]	○	○
Avez-vous entendu des personnes mourir ? [champ_ssoz]	○	○
Avez-vous croisé le regard des terroristes ? [champ_n809]	○	○
Avez-vous vu directement les terroristes ? [champ_qowr]	○	○
Avez-vous perçu la présence des terroristes à proximité de vous ? [champ_bgim]	○	○
Avez-vous dû parler avec les terroristes ? [champ_mlc4]	○	○
Avez-vous été visé par les terroristes ? [champ_sqsb]	○	○
Un terroriste vous a-t-il physiquement touché ? [champ_jn4p]	○	○
Avez-vous simulé votre mort ? [champ_t97h]	○	○
Vous êtes-vous protégé à l'aide de corps ? [champ_eqev]	○	○
Avez-vous eu peur qu'un bruit dénonce votre présence (sonnerie de téléphone, cri, pleurs etc.) ? [champ_a04x]	○	○
Vous êtes-vous retrouvé dans l'impossibilité d'apporter de l'aide ? [champ_k0n8]	○	○
Avez-vous pu aussi apporter de l'aide ? [apporter_aide_bat]	◉	○
Avez-vous eu connaissance que la police allait intervenir ? [police_intervent]	○	○
Avez-vous éprouvé un sentiment ou eu une perception de la situation non listés ici ? [sentiments_bat]	◉	○

Préciser ce sentiment / perception :

Bataclan

Vous étiez :

- A l'intérieur du Bataclan
- A proximité immédiate du bataclan

	Oui	Non
**Avez-vous été confiné ou dû vous mettre à l'abri? [champ_a2wh]**		
**Avez-vous senti l'odeur de la poudre ou une autre odeur particulière provenant des lieux de l'événement ? [champ_t40o]**		
**Avez-vous vu du sang ? [champ_r1bq]**		
**Avez-vous vu des personnes inertes, blessées, mutilées, mortes ou des restes de corps humains ? [champ_jdl3]**		
**Avez-vous touché des personnes blessées ou mortes ? [champ_jcxo]**		
**Avez-vous vu directement quelqu'un être menacé, blessé ou mourir ? [champ_ygw1]**		

Avez-vous éprouvé un sentiment ou eu une perception de la situation non listés ici ?

- Oui
- Non

Laquelle

Avez-vous pu apporter de l'aide ?

- Oui
- Non

Assaut du 18 Novembre

Où étiez-vous lors de l'assaut du 18 novembre à Saint-Denis ?

- ○ Dans l'immeuble du 48 rue de la République
- ○ Dans un immeuble proche

	Oui	Non
	○	○
**Avez-vous été confiné pendant l'assaut ? [champ_rx8c]**	◉	○
**Votre logement a-t-il été endommagé par des tirs ou par l'explosion ? [champ_u927]**	◉	○
**Votre logement est –il devenu inhabitable suite à cet assaut ? [champ_mwab]**	◉	○
**Avez-vous vu du sang ? [champ_yu13]**	◉	○
**Avez-vous vu des personnes inertes, blessées, mutilées, mortes ou des restes de corps humains ? [champ_c05t]**	◉	○
**Avez-vous senti l'odeur de la poudre ou une autre odeur particulière provenant des lieux de l'événement ? [champ_k87e]**	◉	○
**Avez-vous été blessé(e) ? [champ_mh9l]**	◉	○
**Avez-vous reçu des projections de sang ou de restes de corps humains ? [champ_xr1l]**	◉	○
**Avez-vous ressenti le souffle de l'explosion ? [champ_l10d]**	◉	○
**Avez-vous éprouvé un sentiment ou eu une perception de la situation non listés ici ? [champ_wpww]**	◉	○

Laquelle

[ ]

Check-list des symptômes de la réaction de peur initale, extrait du STRS-A3 – Shortness of breath, tremulousness, racing heart and sweating rating scale

Les questions suivantes concernent l’événement que vous avez vécu et qui peut avoir entrainé chez vous une réaction émotionnelle aiguë.

Pourriez-vous, s’il vous plait, répondre à ces quelques questions sur vos réactions **pendant cet événement et quelques heures après** ?

	**Pas du tout** ○	**Légèrement** ○	**Modérément** ○	**Beaucoup** ○	**Extrêmement** ○
**Lors de l’événement, aviez-vous eu des difficultés à respirer ou le souffle coupé ? [champ_yhh2]**	○	○	○	○	○
**Lors de l’événement, aviez-vous ressenti des frissons ou des tremblements des genoux ? [champ_pgok]**	○	○	○	○	○
**Lors de l’évènement, aviez-vous senti des palpitations ou que votre rythme cardiaque s’accélérait ? [champ_z67i]**	○	○	○	○	○
**Lors de l’événement, aviez-vous senti vos mains moites ou des sueurs froides ? [champ_k89g]**	○	○	○	○	○

Questionnaire des expériences de dissociation péritraumatique (PDEQ)

Les questions suivantes concernent l’événement que vous avez vécu et qui peut avoir entrainé chez vous une réaction émotionnelle aiguë. Complétez, s’il vous plaît, les énoncés qui suivent en cochant ce qui correspond au mieux à ce que vous avez ressenti **pendant cet événement et immédiatement après l’événement.** Si une question ne s’applique pas à votre expérience, cochez « Pas du tout vrai ».

	**Pas du tout vrai** ○	**Un peu vrai** ○	**Plutôt vrai** ○	**Très vrai** ○	**Extrêmement vrai** ○
**Il y a eu des moments où j’ai perdu le fil de ce qui se passait, j’étais complètement déconnecté(e) ou d’une certaine façon j’ai senti que je**	○	○	○	○	○

	Pas du tout vrai	Un peu vrai	Plutôt vrai	Très vrai	Extrêmement vrai
	○	○	○	○	○
ne faisais pas partie de ce qui se passait. [champ_k70j]					
Je me suis retrouvé(e) comme en « pilotage automatique », je me suis mis(e) à faire des choses que, je l'ai réalisé plus tard, je n'avais pas activement décidées de faire. [champ_q346]	○	○	○	○	○
Ma perception du temps était changée, les choses avaient l'air de se dérouler au ralenti. [champ_o56o]	○	○	○	○	○
Ce qui se passait me semblait irréel, comme si j'étais dans un rêve, ou au cinéma, ou en train de jouer un rôle. [champ_zan2]	●	○	○	○	○
C'est comme si j'étais le(la) spectateur(trice) de ce qui m'arrivait, comme si je flottais au-dessus de la scène et l'observais de l'extérieur. [champ_gdp9]	○	○	○	○	○
Il y a eu des moments où la perception que j'avais de mon corps était déformée ou changée. Je me sentais déconnecté(e) de mon propre corps, ou bien il me semblait plus grand ou plus petit que d'habitude. [champ_c6qb]	○	○	○	○	○
J'avais l'impression que les choses qui arrivaient aux autres m'arrivaient à moi aussi, comme par exemple être en danger alors que je ne l'étais pas. [champ_u834]	○	○	○	○	○
J'ai été surpris(e) de constater après coup que plusieurs choses s'étaient produites sans que je m'en rende compte, des choses que j'aurais habituellement remarquées. [champ_v4ed]	○	○	○	○	○

	Pas du tout vrai	Un peu vrai	Plutôt vrai	Très vrai	Extrêmement vrai
	○	○	○	○	○
**J'étais confus(e) ; c'est-à-dire que par moment j'avais de la difficulté à comprendre ce qui se passait vraiment. [champ_oa6x]**	○	○	○	○	○
**J'étais désorienté(e) ; c'est-à-dire que par moment j'étais incertain(e) de l'endroit où je me trouvais, ou de l'heure qu'il était. [champ_o2y4]**	○	○	○	○	○

Pourriez-vous évaluer sur une échelle allant de 0 à 10 jusqu'à quel point il vous semble avoir été exposé(e) à l'événement ? De 0 = je n'ai pas réellement été exposé à 10 = je fais partie des personnes les plus exposées

○ 0
○ 1
○ 2
○ 3
○ 4
○ 5
○ 6
○ 7
○ 8
○ 9
○ 10

Si vous ne vous êtes pas reconnu(e) dans les questions précédentes, pouvez-vous exprimer en quoi vous vous êtes senti exposé(e) (bouleversé(e)) ?

Vos blessures physiques et leurs conséquences

Avez-vous été blessé(e) physiquement lors des attentats ?

- Oui
- Non

Vos soins ont nécessité une hospitalisation initiale (en urgence) qui s'est prolongée plus d'une semaine

- Oui
- Non

Vos soins ont nécessité une hospitalisation initiale (en urgence) de moins d'une semaine

- Oui
- Non

Vos soins ont nécessité une hospitalisation programmée/différée

- Oui
- Non

Et maintenant pourriez-vous nous informer sur ce qui s'est passé pendant l'hospitalisation (première hospitalisation si plusieurs) pour votre ou vos blessures physiques

Avez-vous bénéficié d'un soutien psychologique par un psychiatre ou un psychologue pendant l'hospitalisation ?

- Oui
- Non

Pouvez-vous nous dire combien de fois avez-vous eu de tels entretiens ?

- Un
- Entre 2 et 5
- Entre 6 et 10
- Plus de 10

La personne avec laquelle vous avez eu le plus d'entretiens était

- Un psychologue
- Un psychiatre
- Autre
- Ne sais pas

Pouvez-vous nous donner votre degré de satisfaction du soutien psychologique que vous avez reçu pendant l'hospitalisation sur une échelle de 1 à 5 ?

Auriez-vous une remarque ou une proposition à faire à ce sujet ?

Après votre hospitalisation, avez-vous continué un suivi psychologique avec une personne qui vous a pris en charge psychologiquement dans cet hôpital ?

- Oui
- Non

Séquelles des blessures physiques

Séquelles à la tête

Avez-vous des problèmes d'auditions (perte auditive, sifflement, bourdonnement dans les oreilles) qui sont apparus du fait des attaques ?

- Oui
- Non

Avez-vous des problèmes de vue qui sont apparus du fait des attaques ?

- Oui
- Non

Avez-vous été touché ailleurs à la tête ?

- Oui
- Non

Avez-vous des douleurs persistantes à la tête (migraines, maux de têtes etc.) ?

- Oui
- Non

Gene locomotrice ou d'un membre

Avez-vous des difficultés à vous déplacer depuis les attaques ?

- Oui
- Non

Y a-t-il encore au moins une opération de prévue ?

- Oui
- Non

Avez-vous dû être opéré pour un problème lié à cette mobilité ?

○ Oui

○ Non

Avez-vous des difficultés à utiliser un ou des membres supérieurs depuis les attentats ?

○ Oui

○ Non

Prenez-vous des médicaments pour ces douleurs ?

○ Oui

○ Non

Souffrez-vous d'une autre séquelle somatique ?

○ Oui

○ Non

Préjudice esthétiques

Est-ce que les attaques ont changé votre apparence au point d'interférer dans vos relations avec les autres ? (1 pas du tout à 5 énormément ou presque tout le temps)

○ 1

○ 2

○ 3

○ 4

○ 5

Souhaiteriez-vous oublier que votre apparence a changé ? (1 pas du tout à 5 énormément ou presque tout le temps)

○ 1

○ 2

○ 3

○ 4

○ 5

Pensez-vous que des membres de votre famille ou des amis sont gênés d'être avec vous en raison de votre apparence ? (1 pas du tout à 5 énormément ou presque tout le temps)

○ 1

○ 2

- 3
- 4
- 5

Avez-vous l'impression que les gens ne veulent plus vous toucher ? (1 pas du tout à 5 énormément ou presque tout le temps)

- 1
- 2
- 3
- 4
- 5

Expertise médicale des blessures physiques

Avez-vous déjà passé une expertise médicale spécialisée pour évaluer les lésions et votre handicap ?

- Oui
- Non

Avez-vous eu besoin de demander conseil à un médecin sur les risques de contamination du fait d'avoir été contact avec du sang d'une autre personne ?

- Oui
- Non
- Non concerné

Avez-vous fait appel aux services d'une Maison Départementale des Personnes Handicapées (MDPH) ?

- Oui
- Non
- Non concerné(e)

Concernant vos proches

Avez-vous un ou plusieurs proches qui ont été directement menacés par les terroristes et/ou blessés physiquement ou psychiquement lors de ces attentats ?

- ( ) Oui
- (•) Non

Étai(en)t-ce

- [ ] Enfant
- [ ] Un(e) conjoint(e)
- [ ] Grand parent
- [ ] Un arrière grand parent
- [ ] Un petit enfant
- [ ] Un arrière petit enfant
- [ ] Un parent
- [ ] Un autre membre de votre famille
- [ ] Un ami
- [ ] Un(e) collègue
- [ ] Une connaissance
- [ ] Autre

Étai(en)t-il(s)

- ( ) Avec vous
- ( ) Sur un autre lieu d'attentat

Avez-vous perdu un ou plusieurs proches lors de ces attentats ?

- (•) Oui
- ( ) Non

Quel était votre lien avec la ou les personnes décédées dans les attentats ? (plusieurs réponses possibles)

- [ ] Enfant
- [ ] Un(e) conjoint(e)
- [ ] Grand parent
- [ ] Un arrière grand parent
- [ ] Un petit enfant

- [ ] Un arrière petit enfant
- [ ] Un parent
- [ ] Un autre membre de votre famille
- [ ] Un ami
- [ ] Un(e) collègue
- [ ] Une connaissance
- [ ] Autre

Etai(en)t-il(s) avec vous au moment où ils sont morts ? (plusieurs réponses possibles)

- [ ] Oui
- [ ] Non

Annonce du décès

Comment avez-vous appris que cette personne ou ces personnes étaient mortes ? (plusieurs réponses possibles)

- [ ] Vous l'avez compris sur le moment
- [ ] Vous avez appris la mort après les attentats

Jusqu’à quel jour avez-vous été confronté à une incertitude concernant le décès de votre ou vos proches ? (plusieurs réponses possibles)

- [ ] Vendredi 13 novembre
- [ ] Samedi 14 novembre
- [ ] Dimanche 15 novembre
- [ ] Lundi 16 novembre
- [ ] Mardi 17 novembre
- [ ] Mercredi 18 novembre
- [ ] Plus tard

Pendant ce délais, avez-vous recherché votre/vos proche(s) ? (plusieurs réponses possibles)

- [ ] Oui
- [ ] Non

Où étiez-vous quand vous avez appris le décès de vos/votre proche(s) ? (plusieurs réponses possibles)

- [ ] Sur les lieux des attentats

- ☐ À l’hôpital
- ☐ À l’école militaire
- ☐ À l’institut médico-légal
- ☐ À la mairie du XIème
- ☐ À la mairie du Xème (ou école Parmentier)
- ☐ Annonce par téléphone à domicile
- ☐ Annonce par une personne à domicile
- ☐ Autre

Qui vous a appris le(s) décès? (plusieurs réponses possibles)

- ☐ Un officier de police judiciaire
- ☐ Un professionnel de santé
- ☐ Un membre de la famille ou un ami proche
- ☐ Les media
- ☐ La CIAV
- ☐ Les réseaux sociaux
- ☐ Autre

Avez-vous la sensation que dans les instants qui ont suivi l’annonce du décès votre confort et votre intimité ont été respectés ?

- ☐ Oui
- ☐ Non

Pour faire face à l’annonce du décès, vous étiez

- ☐ Seul(e)
- ☐ Accompagné(e) par un ou des proches

Avez-vous dû annoncer le décès à l’entourage proche de la victime ?

- ☐ Oui
- ☐ Non

Avez-vous été mis(e) en position de devoir annoncer ce qui s'était passé à un ou des enfants ?

- ☐ Oui
- ☐ Non

Avez-vous des précisions que vous souhaiteriez apporter sur les conditions de cette annonce ?

○ Oui

○ Non

Constat du décès

Avez-vous pu voir le corps de votre ou de vos proches ?

☐ Oui

☐ Non

Avez-vous été confronté à des images ou informations bouleversantes concernant la mort d'un de vos proches dans les médias ou sur internet ?

○ Oui

○ Non

Si vous ne vous êtes pas reconnu(e) dans les questions précédentes, pouvez-vous exprimer en quoi vous vous êtes senti exposé(e) (bouleversé(e)) ?

Ce que vous éprouvez psychologiquement

The Posttraumatic Stress Disorder Checklist (PCL-5)

Voici une liste de problèmes que les gens éprouvent parfois suite à une expérience vraiment stressante (ici il est question de ces attentats et de votre vécu de ces attentats). Veuillez lire chaque énoncé attentivement et cocher pour indiquer dans quelle mesure ce problème vous a affecté dans le **dernier mois**.

Dans le dernier mois, dans quelle mesure avez-vous été affecté par

	Pas du tout ○	Un peu ○	Moyennement ○	Souvent ○	Extrêmement ○
**Des souvenirs répétés, pénibles et involontaires de l'expérience stressante ? [champ_lpcl5_1]**	○	○	○	○	○
**Des rêves répétés et pénibles de l'expérience stressante? [champ_lpcl5_2]**	○	○	○	○	○
**Se sentir soudainement comme si l'expérience stressante recommençait (comme si vous la viviez de nouveau)? [champ_lpcl5_3]**	○	○	○	○	○
**Être bouleversé(e) lorsque quelque chose vous rappelle l'expérience stressante? [champ_lpcl5_4]**	○	○	○	○	○
**Réagir physiquement lorsque quelque chose vous rappelle l'expérience stressante (p. ex., avoir le coeur qui bat très fort, du mal à respirer, ou avoir des sueurs)? [champ_lpcl5_5]**	○	○	○	○	○
**Éviter souvenirs, pensées ou sentiments en lien avec l'expérience stressante? [champ_lpcl5_6]**	○	○	○	○	○
**Éviter les personnes et les choses qui vous rappellent l'expérience stressante (p. ex., des gens, des lieux, des conversations, des activités, des objets, ou des situations)? [champ_lpcl5_7]**	○	○	○	○	○
**Avoir du mal à vous rappeler d'éléments importants de l'expérience stressante? [champ_lpcl5_8]**	○	○	○	○	○

	Pas du tout	Un peu	Moyennement	Souvent	Extrêmement
	○	○	○	○	○
**Avoir des croyances négatives sur vous-même, les autres ou sur le monde (p. ex., avoir des pensées telles que : je suis mauvais, il y a quelque chose qui cloche sérieusement chez moi, nul n'est digne de confiance, le monde est un endroit complètement dangereux)? [champ_lpcl5_9]**	○	○	○	○	○
**Vous blâmer ou blâmer les autres pour la survenue de l'expérience stressante ou ce qui est arrivé par la suite? [champ_lpcl5_10]**	○	○	○	○	○
**Avoir des sentiments négatifs intenses tels que peur, horreur, colère, culpabilité, ou honte? [champ_lpcl5_11]**	○	○	○	○	○
**Perdre de l'intérêt pour des activités que vous aimiez auparavant? [champ_lpcl5_12]**	○	○	○	○	○
**Vous sentir distant ou coupé des autres? [champ_lpcl5_13]**	○	○	○	○	○
**Avoir du mal à éprouver des sentiments positifs (p. ex., être incapable de ressentir la joie ou de l'amour envers vos proches)? [champ_lpcl5_14]**	○	○	○	○	○
**Être irritable, avoir des bouffées de colère, ou agir agressivement? [champ_lpcl5_15]**	○	○	○	○	○
**Prendre des risques inconsidérés ou encore avoir des conduites qui pourraient vous mettre en danger ? [champ_lpcl5_16]**	○	○	○	○	○
**Être en état de 'super-alerte', vigilant ou sur vos gardes? [champ_lpcl5_17]**	○	○	○	○	○
**Sursauter facilement? [champ_lpcl5_18]**	○	○	○	○	○
**Avoir du mal à vous concentrer? [champ_lpcl5_19]**	○	○	○	◉	○

	Pas du tout	Un peu	Moyennement	Souvent	Extrêmement
	○	○	○	○	○
**Avoir du mal à trouver ou garder le sommeil? [champ_lpcl5_20]**	○	○	○	○	○

Pourriez-vous chiffrer de 0 à 4 l'intensité avec laquelle vous avez ressenti les problèmes que vous venez d'exprimer pour chacun des mois suivant l'événement : 0 = rien du tout ; 1 = faible ; 2 = notable ; 3 = forte ; 4 = majeure

	0	1	2	3	4
	○	○	○	○	○
**Novembre [diif_nov]**	○	○	○	○	○
**Décembre [diff_dec]**	○	○	○	○	●
**Janvier [diff_janv]**	○	○	●	○	○
**Février [diff_fev]**	○	○	○	○	○
**Mars [diff_mars]**	○	○	○	○	○
**Avril [diff_avril]**	○	○	○	○	○
**Mai [diff_mai]**	○	●	○	○	○
**Juin [diff_juin]**	○	○	○	○	○
**Juillet [champ_ahfq]**	○	○	○	○	○
**Août [champ_vfsj]**	○	○	○	○	○
**Septembre [champ_nqiy]**	○	○	○	○	○

Est-ce que ces difficultés rendent vos relations avec votre famille plus difficiles ?

● Oui

○ Non

Est-ce que ces difficultés vous posent des problèmes pour vous entendre avec vos amis ?

● Oui

○ Non

Est-ce que ces difficultés vous posent des problèmes pour bien travailler ?

● Oui

- [ ] Non

Est-ce que ces difficultés vous posent des problèmes pour votre niveau général de fonctionnement dans la vie ?

- [ ] Oui
- [x] Non

Echelle HAD : **Hospital anxiety and depression scale**

Instructions : ce questionnaire a été conçu de façon à vous permettre d'exprimer ce que vous éprouvez sur le plan émotif. Lisez chaque série de questions et choisissez la réponse qui exprime le mieux ce que vous avez éprouvé **au cours des 7 jours qui viennent de s'écouler.** Ne vous attardez pas sur la réponse à faire : votre réaction immédiate à chaque question fournira probablement une meilleure indication de ce que vous éprouvez qu'une réponse longuement méditée.
DANS LES 7 DERNIERS JOURS QUI VIENNENT DE S'ECOULER

Je me sens tendu(e) ou énervé(e)

- [x] La plupart du temps
- [ ] Souvent
- [ ] De temps en temps
- [ ] Jamais

Je prends plaisir aux mêmes choses qu'autrefois

- [ ] Oui, tout autant qu'avant
- [ ] Pas autant
- [ ] Un peu seulement
- [ ] Presque plus

J'ai une sensation de peur comme si quelque chose d'horrible allait m'arriver

- [ ] Oui, très nettement
- [ ] Oui, mais ce n'est pas trop grave
- [ ] Un peu, mais cela ne m'inquiète pas
- [ ] Pas du tout

Je ris facilement et vois le bon côté des choses

- [ ] Autant que par le passé
- [ ] Plus autant qu'avant

- Vraiment moins qu'avant
- Plus du tout

Je me fais du souci

- Très souvent
- Assez souvent
- Occasionnellement
- Très occasionnellement

Je suis de bonne humeur

- Jamais
- Rarement
- Assez souvent
- La plupart du temps

Je peux rester tranquillement assis(e) à ne rien faire et me sentir décontracté(e)

- Oui, quoi qu'il arrive
- Oui, en général
- Rarement
- Jamais

J'ai l'impression de fonctionner au ralenti

- Presque toujours
- Très souvent
- Parfois
- Jamais

J'éprouve des sensations de peur et j'ai l'estomac noué

- Jamais
- Parfois
- Assez souvent
- Très souvent

Je ne m'intéresse plus à mon apparence

- Plus du tout
- Je n'y accorde pas autant d'attention que je devrais
- Il se peut que je n'y fasse plus autant attention

- ○ J’y prête autant d’attention que par le passé

J’ai la bougeotte et n’arrive pas à tenir en place

- ○ Oui, c’est tout à fait le cas
- ○ Un peu
- ○ Pas tellement
- ○ Pas du tout

Je me réjouis d’avance à l’idée de faire certaines choses

- ○ Autant qu’avant
- ○ Un peu moins qu’avant
- ○ Bien moins qu’avant
- ○ Presque jamais

J’éprouve des sensations soudaines de panique

- ○ Vraiment très souvent
- ○ Assez souvent
- ○ Pas très souvent
- ○ Jamais

Je peux prendre plaisir à un bon livre ou à une bonne émission de radio ou de télévision

- ○ Souvent
- ○ Parfois
- ○ Rarement
- ○ Très rarement

Pensées suicidaires

Les souffrances que vous avez endurées depuis le jour des attentats, vous ont-elles conduit à avoir des pensées suicidaires ?

- ◉ Oui
- ○ Non

A quelle période sont-elles apparues ? (merci d’indiquer le mois d’apparition puis chaque mois où elles ont été présentes)

- ☐ Novembre
- ☐ Décembre

- ☐ Janvier
- ☐ Février
- ☐ Mars
- ☐ Avril
- ☐ Mai
- ☐ Juin
- ☐ Juillet
- ☐ Aout
- ☐ Septembre

A l'aide des repères suivants, indiquez le degré de développement maximal de vos pensées suicidaires ?

- ○ Idées furtives
- ○ Idées fréquentes sans intention
- ○ Intention suicidaire
- ○ Programmation d'un geste suicidaire
- ○ Tentative de suicide interrompue
- ○ Tentative de suicide

Avez-vous consulté un médecin ou un psychologue en raison de l'apparition de ces idées de suicide ?

- ○ Oui
- ○ Non

Dans l'année précédant les attentats, aviez-vous présenté des pensées suicidaires ?

- ○ Oui
- ◉ Non

Inventaire de deuil compliqué – échelle de Prigerson

Cochez la réponse qui décrirait le mieux ce que vous ressentez en ce moment

	**Jamais**	**Rarement**	**Parfois**	**Souvent**	**Toujours**
	○	○	○	○	○
**Je pense tellement à la personne décédée qu'il m'est**	○	○	○	○	○

	Jamais	Rarement	Parfois	Souvent	Toujours
	○	○	○	○	○
difficile de faire les choses normalement [champ_v35j]					
Les souvenirs de la personne décédée me bouleversent [champ_k5d4]	○	○	○	○	○
Il est difficile d'accepter la mort de cette personne [champ_k6jp]	○	○	○	○	○
Je me languis de la personne décédée [champ_m2dd]	○	○	○	○	○
Je me sens attiré(e) vers les lieux ou les choses qui me font penser à la personne décédée [champ_kn55]	○	○	○	○	○
Je suis en colère à propos de la mort de cette personne [champ_t90k]	○	○	○	○	○
Je refuse de croire ce qui est arrivé [champ_b043]	○	○	○	○	○
Je suis sidéré(e) ou stupéfait(e) à propos de ce qui est arrivé [champ_c2dj]	○	○	○	○	○
Depuis le décès de cette personne il est difficile pour moi de faire confiance aux gens [champ_j4cv]	○	○	○	○	○
Je me sens comme si je ne pouvais plus prendre soin des autres personnes ou je me sens distant(e) par rapport aux personnes qui me sont chères [champ_a75u]	○	○	○	○	○
Je ressens le même type de douleur ou de symptômes que la personne décédée [champ_j9d1]	○	○	○	○	○
Je fais en sorte d'éviter ce qui me rappelle la personne décédée [champ_lplj]	○	○	○	○	○

	Jamais	Rarement	Parfois	Souvent	Toujours
	○	○	○	○	○
**Je trouve la vie dénuée de sens sans la personne décédée [champ_y5ly]**	○	○	○	○	○
**J'entends la voix de la personne décédée [champ_fww1]**	○	○	○	○	○
**Je vois réellement la personne décédée en face de moi [champ_v9s1]**	○	○	○	○	○
**Je trouve injuste de devoir continuer à vivre alors que cette personne est morte [champ_d77o]**	○	○	○	○	○
Je ressens de l'amertume par rapport au décès de cette personne [champ_l93j]	○	○	○	○	○
**J'envie les personnes qui n'ont pas perdu une personne proche [champ_r5u0]**	○	○	○	○	○
**Je me sens seule(e) la plupart du temps depuis le décès de cette personne [champ_r8cw]**	○	○	○	○	○

Le soutien psychologique que vous avez reçu

Avant d'avoir pu regagner votre lieu d'hébergement

Avez-vous des souvenirs de ce que vous avez vécu entre les suites immédiates de l'événement et le moment où vous avez regagné votre lieu d'hébergement ?

- (•) Oui
- ( ) Non

Avez-vous le souvenir d'avoir reçu un soutien ou une aide ?

- ( ) Oui
- (•) Non

Cette aide ou ce soutien était apporté par un ou plusieurs tiers (une personne non secouriste ou professionnelle de santé ou force de l'ordre)

- ( ) Oui
- ( ) Non

Cette aide ou ce soutien était apporté par : Des personnes de la Croix Rouge française, de la Protection civile de Paris, ou de l'ordre de Malte

- ( ) Oui
- ( ) Non

Cette aide ou ce soutien était apporté par des personnels de la police

- ( ) Oui
- ( ) Non

Cette aide ou ce soutien était apporté par

- [ ] Des personnels non identifiés
- [ ] Pompiers
- [ ] SAMU
- [ ] CUMP
- [ ] Autre personnel de soin

SAMU / **Des personnels non identifiés/ Pompiers/CUMP/ Autre personnel de soin**

Pourriez-vous dire dans quel(s) lieu(x)?

- [ ] Sur le lieu de l'événement
- [ ] Dans la rue
- [ ] A l'Hôtel Dieu
- [ ] Dans un autre hôpital

- [ ] Dans une mairie
- [ ] Dans une école
- [ ] Au commissariat
- [ ] Ailleurs
- [ ] Je ne sais pas

Préciser

[ ]

Comment êtes-vous rentré(e) en contact ?

- ○ À votre initiative
- ○ La(les) personnes est (sont) venue à vous
- ○ Vous avez été orienté(e) vers ces professionnels

Avez-vous bénéficié d'un ou plusieurs entretiens ?

- ○ Oui
- ○ Non

Avez-vous bénéficié d'une autre forme de soutien (café, couverture...) ?

- ○ Oui
- ○ Non

Vous a-t-on prescrit des médicaments ?

- ○ Oui
- ○ Non

Vous a-t-on prescrit un arrêt de travail ?

- ○ Oui
- ○ Non

Vous a-t-on remis une liste des structures de prises en charges psychologiques ?

- ○ Oui
- ○ Non

Vous a-t-on remis un certificat médical initial ?

- ○ Oui
- ○ Non

Est-ce que quelqu'un a pris vos coordonnées ?

- ○ Oui
- ○ Non

Êtes-vous satisfait de votre interaction avec cette personne qui vous a pris en charge ?

- ○ 1
- ○ 2
- ○ 3

- ○ 4
- ○ 5

Avant d’avoir pu regagner votre lieu d’hébergement, avez-vous été hospitalisé(e) pour des raisons psychologiques ?

- ○ Oui
- ◉ Non

Était-ce :

- ○ À votre initiative personnelle
- ○ À l’initiative de votre entourage
- ○ Sur conseil de votre hiérarchie professionnelle ou médecine de prévention
- ○ Sur conseil d’un psychologue
- ○ Sur prescription de votre médecin généraliste
- ○ Sur prescription de votre psychiatre
- ○ Sur proposition d’une association d’aide aux victimes
- ○ Sur proposition d’une association de victimes
- ○ Autre

Etait-ce

- ○ En urgence
- ○ De façon programmée

Pouvez-vous nous dire pour quel problème psychologique?

- ☐ Troubles du sommeil
- ☐ Syndrome dépressif
- ☐ Tentative ou risque de suicide
- ☐ Troubles anxieux
- ☐ Consommation accrue de substances psychoactives (alcool, tabac, drogue, médicaments
- ☐ État de stress post-traumatique
- ☐ Deuil compliqué
- ☐ Autre

A quelle date

Combien de jours avez-vous été hospitalisé ?

[ ]

Vous a-t-on à l’issue orienté ?

- ○ Oui
- ○ Non

Vers quel service ?

Considérez-vous durant cette période d'hospitalisation que vous avez reçu une prise en charge médico-psychologique appropriée de la part des professionnels pour faire face à l'ensemble des stress que vous avez vécu suite aux événements ?

- Non pas du tout
- Non pas vraiment
- Oui un peu
- Oui tout à fait
- Ne sait pas

Auriez-vous une remarque à faire au sujet de la prise en charge médico-psychologique durant cette hospitalisation ?

Considérez-vous durant cette période (avant votre retour dans un lieu d'hébergement) que vous avez reçu une aide psychologique appropriée de la part des professionnels pour faire face à l'ensemble des stress que vous avez vécu suite aux événements ?

- Non pas du tout
- Non pas vraiment
- Oui un peu
- Oui tout à fait
- Ne sait pas

Auriez-vous une remarque à faire au sujet de l'aide psychologique durant cette période ?

Depuis les événements (après avoir regagné votre lieu d'hébergement pour les personnes sur les lieux au moment des attaques)

Êtes-vous allé(e) dans un de ces lieux d'accueil mis en place juste après les attentats ?

- ☐ Mairie du 10e/école Parmentier
- ☐ Mairie du 11e
- ☐ Mairie de Saint-Denis ou centre municipal de santé rue du Cygne
- ☐ Institut médico-légal

- [ ] Ecole Militaire
- [ ] Hôtel-Dieu
- [ ] Autre

Préciser

Mairie du 11e/ Mairie de Saint-Denis ou centre municipal de santé rue du Cygne/ Institut médico-légal/Hôtel-Dieu/ Ecole Militaire/Autre

Avez-vous bénéficié d'un soutien psychosocial des secouristes associatifs (Croix Rouge, Protection civile de Paris)?

- ○ Oui
- ○ Non

Avez-vous bénéficié d'un soutien psychosocial des professionnels de santé (CUMP, service de santé des armées, etc) ?

- ○ Oui
- ○ Non

A quel(s) moment(s) ?

- [ ] Dans les 48 premières heures
- [ ] Entre 2 et 7 jours après
- [ ] Après la première semaine
- [ ] Je ne sais plus

Comment êtes-vous rentré(e) en contact avec les personnes qui vous ont pris en charge ?

- ○ À votre initiative
- ○ La(les) personnes est (sont) venue à vous
- ○ Vous avez été orienté(e) vers ces professionnels

Avez-vous bénéficié d'un ou plusieurs entretiens ?

- ○ Oui
- ○ Non

Avez-vous bénéficié d'une prescription médicamenteuse ?

- ○ Oui
- ○ Non

Avez-vous bénéficié d'un arrêt maladie ?

- ○ Oui
- ○ Non

Vous a-t-on remis une liste des structures de prises en charges psychologiques ?

- ○ Oui
- ○ Non

Avez-vous bénéficié d’un certificat médical initial ?

- Oui
- Non

Est-ce que quelqu’un a pris vos coordonnées ?

- Oui
- Non

Êtes-vous satisfait de votre interaction avec cette institution ou cette personne ?

- 1
- 2
- 3
- 4
- 5

A l'école militaire
Avez-vous bénéficié d'un soutien psychosocial
D'une association de victimes (FENVAC, AVFT)

- ○ Oui
- ○ Non

D'une association d'aide aux victimes (Paris Aide aux Victime, Inavem, autre association du réseau Inavem etc.)

- ○ Oui
- ○ Non

D'une association d'aide aux victimes ou de victime mais je ne me rappelle pas laquelle

- ○ Oui
- ○ Non

En dehors des lieux cités précédemment êtes-vous allé voir ou avez-vous été reçu(e) par une (des) personne(s) d'un organisme, d'une association, d'un cabinet libéral pour vos difficultés psychologiques ?

○ Oui

◉ Non

A quel organisme, association ou institution appartenai(en)t-il(s) ?

☐ Services d'urgence d'un hôpital

☐ Consultation spécialisée en hôpital pour le psychotraumatisme

☐ Consultation médico psychologique (CMP)

☐ CUMP

☐ Consultation spécialisée en secteur libéral

☐ Association d'Aide aux victimes INAVEM (ex. Paris aide aux victimes, ADAVIP 92)

☐ Association de victimes (AFVT –FENVAC)

☐ Ose – Œuvre de secours aux enfants

☐ Médecin traitant

☐ Je ne sais pas

☐ Autre

Préciser/ ………./

Services d'urgence d'un hôpital/ Consultation spécialisée en hôpital pour le psychotraumatisme/ Consultation médico psychologique (CMP)/ CUMP/Consultation spécialisée en secteur libéral/ Association d'Aide aux victimes INAVEM (ex. Paris aide aux victimes, ADAVIP 92)/ Association de victimes (AFVT –FENVAC)/ Ose – Œuvre de secours aux enfants/ Médecin traitant/ Je ne sais pas/ Autre

Comment êtes-vous rentré(e) en contact ?

○ À votre initiative

○ La(les) personnes est (sont) venue à vous

○ Vous avez été orienté(e) vers ces professionnels

La(les) personne(s) qui vous a(ont) soutenu étai(en)t-elle(s) ?

☐ Psychologue

☐ Psychiatre

☐ Infirmier

☐ Je ne sais pas

☐ Médecin

☐ Autre

L'entretien était-il

○ Partagé avec d'autres personnes ?

○ Individuel (personne à personne) ?

L'institution ou la personne vous a-t-il(elle) orienté pour une prise en charge ?

○ Oui

○ Non

☐ Depuis les événements, avez-vous été hospitalisé pour des difficultés psychologiques :

○ Oui

◉ Non

Était-ce

☐ À votre initiative personnelle

☐ À l'initiative de votre entourage

☐ Sur conseil de votre hiérarchie professionnelle ou médecine de prévention

☐ Sur conseil d'un psychologue

☐ Sur prescription de votre médecin généraliste

☐ Sur prescription de votre psychiatre

☐ Sur proposition d'une association d'aide aux victimes

☐ Sur proposition d'une association de victimes

☐ Autre

Était-ce

○ En urgence

○ De façon programmée

Pouvez-vous nous dire pour quel problème psychologique?

☐ Troubles du sommeil

☐ Syndrome dépressif

☐ Tentative ou risque de suicide

☐ Troubles anxieux

☐ Consommation accrue de substances psychoactives (alcool, tabac, drogue, médicaments

☐ État de stress post-traumatique

☐ Deuil compliqué

☐ Autre

A quelle date

Combien de jours avez-vous été hospitalisé ?

Vous a-t-on à l'issue orienté ?

- ○ Oui
- ○ Non

Considérez-vous durant cette période d'hospitalisation que vous avez reçu une prise en charge médico-psychologique appropriée de la part des professionnels pour faire face à l'ensemble des stress que vous avez vécu suite aux événements ?

- ○ Non pas du tout
- ○ Non pas vraiment
- ○ Oui un peu
- ○ Oui tout à fait
- ○ Ne sait pas

Auriez-vous une remarque à faire au sujet de la prise en charge médico-psychologique durant cette hospitalisation ?

Depuis les événements, avez-vous engagé des soins médico-psychologiques réguliers ?

- ● Oui
- ○ Non

Était ce

- [ ] À votre initiative personnelle
- [ ] Sur conseil de votre entourage
- [ ] Sur conseil de votre hiérarchie professionnelle ou médecine de prévention
- [ ] Sur conseil d'un psychologue
- [ ] Sur prescription de votre médecin généraliste
- [ ] Sur prescription de votre psychiatre
- [ ] Sur proposition d'une association d'aide aux victimes
- [ ] Sur proposition d'une association de victimes
- [x] Autre

Préciser

Était ce

- [ ] Avec un psychiatre de secteur public
- [ ] Avec un psychiatre libéral
- [ ] Avec un psychiatre d'une consultation spécialisée en psycho-traumatisme
- [ ] Avec un psychologue de secteur public
- [ ] Avec un psychologue libéral
- [ ] Avec un psychologue d'une consultation spécialisée en psycho-traumatisme
- [ ] Avec un psychologue d'une association d'aide aux victimes
- [ ] Avec un psychothérapeute
- [ ] Avec un psychanalyste
- [ ] Avec un psychologue ou psychiatre des armées
- [x] Autre

Préciser

Savez quelle forme de thérapie vous avez engagé ?

- [ ] Une thérapie psycho-dynamique ou psychanalytique
- [ ] Une thérapie comportementale et cognitive (TCC),
- [ ] Paris MEM (propanolol)
- [ ] Une approche par hypnose
- [ ] Une approche par EMDR (Eye Movement Desensitization and Reprocessing
- [ ] Ne sait pas
- [x] Autre

Préciser

À quelle date avez-vous engagé une prise en charge médicopsychologique régulière ?

Votre suivi est-il en cours ?

- ○ Oui
- ○ Non

Y a-t-il eu une (des) interruption(s)puis reprise(s) de votre suivi ?

- ○ Oui
- ○ Non

Avez-vous changé (une ou plusieurs fois) de consultant ?

- ○ Oui
- ○ Non

Pensez-vous que cette prise en charge vous a été ou vous est encore utile ? (1 = pas du tout à 5 = tout à fait)

- ○ 1

○ 2

○ 3

○ 4

○ 5

En êtes-vous satisfait ? (1 = pas du tout à 5 = tout à fait)

○ 1

○ 2

○ 3

○ 4

○ 5

Auriez-vous une remarque à faire au sujet de ces soins réguliers ?

Depuis les événements, avez-vous engagé des soins médico-psychologiques réguliers ?

○ Oui

● Non

Cochez la case (les cases) correspondant à votre situation

☐ On ne vous l’a pas proposé

☐ On vous l’a proposé mais vous n’en ressentiez pas le besoin

☐ Vous ne saviez pas que c’était possible

☐ On vous l’a proposé mais vous ne vouliez pas parler/ce n’était pas le bon moment

☐ Les modalités proposées ne vous convenaient pas

☐ vous n’avez pas trouvé de professionnel disponible

☐ Vous avez eu une mauvaise expérience

☐ Vous étiez déjà suivi

☐ Vous ne saviez pas vers qui vous tourner

☐ Pour des raisons de coût financier

☐ Vous n'en ressentez pas le besoin

☑ Autre raison

Préciser : /……../

Depuis les événements, vous a-t-on prescrit des médicaments pour des difficultés relatives :

Au sommeil

◉ Oui

○ Non

À l'angoisse, au stress

◉ Oui

○ Non

Depuis quand ?

Les prenez-vous toujours ?

○ Oui

○ Non

À la dépression

◉ Oui

○ Non

Depuis les événements avez-vous consulté des informations sur internet en lien avec vos difficultés psychologiques (conseil médical, application de santé connectée)

◉ Oui

○ Non

Considérez-vous que l'information que vous y avez recueillie était appropriée pour faire face à l'ensemble des stress que vous avez vécu suite aux événements ? (1 = pas du tout à 5 = tout à fait)

○ 1

○ 2

○ 3

○ 4

○ 5

○ Ne sait pas

Auriez-vous une remarque à faire au sujet de ces informations on-line ?

Au sujet de certains de vos contacts téléphoniques liés aux événements

Depuis les événements avez-vous été recontacté au téléphone par des professionnels qui vous ont pris en charge dès les premiers moments ?

- (x) Oui
- ( ) Non

Par qui ?

Depuis les événements avez-vous été recontacté au téléphone par des professionnels qui vous ont pris en charge par la suite?

- ( ) Oui
- (x) Non

Par qui ?

Depuis les événements avez-vous appelé au téléphone pour des problèmes psychologiques liés aux évènements ?

- (x) Oui
- ( ) Non

Était-ce pour rechercher des informations?

- ( ) Oui
- (x) Non

Qui avez-vous contacté ?

Considérez-vous que l'info que vous avez recueillie était appropriée pour faire face à l'ensemble des stress que vous avez vécu suite aux événements ? (1 = pas du tout à 5 = tout à fait)

- ( ) 1
- ( ) 2
- ( ) 3
- ( ) 4

- [ ] 5
- [ ] Ne sait pas

Auriez-vous une remarque à faire au sujet de ces contacts téléphoniques ?

Était-ce pour rechercher du soutien ou de l’aide ?

- [ ] Oui
- [x] Non

Si vous êtes en possession de l'attestation de prise en charge et de dispense d'avance de frais des proches parents des victimes d'actes de terrorisme délivrée par votre caisse de sécurité sociale, l'avez-vous utilisé ?

- [ ] Oui
- [x] Non

Avez-vous trouvé cela utile?

- [ ] Oui
- [ ] Non

Avez-vous des remarques ou suggestions à faire à propos de ce dispositif?

Avez-vous ressenti le besoin de participer à une association de personnes endeuillées ?

- [ ] Oui
- [x] Non
