# Supplementary material for: Use of mental health supports by civilians exposed to the November 2015 terrorist attacks in Paris
Source: BMC Health Serv Res. 2020 Oct 20;20:959. doi: 10.1186/s12913-020-05785-3 (PMC7574168; doi:10.1186/s12913-020-05785-3)
Supplement: Supplementary file 2 — Additional file 2. Translation of the relevant variables used in the civilian version of the French web-based questionnaire for Phase 1 of the ESPA_ 13_November study. Additional file 2 is the English language clean-copy version of the questions from the web-interview guide used in the construction of the variables analysed for this article. [file 12913_2020_5785_MOESM2_ESM.docx]

Additional file 2: Translation of the relevant variables used in the civilian version of the French web-based questionnaire for Phase 1 of the ESPA_ 13_ November study

NB: We present here an excerpt from the introductory page of the web-based questionnaire for the participants of the study. In the following pages, only the questions used to construct the variables used for the analysis are presented.

Brève description des thèmes abordés.

Brief description of the topics covered

[**Votre situation actuelle**](https://impacts_epidemio.preprod.voozanoo.net/espa_13_novembre/)

**Your current situation**

Ces données sont classiquement recueillies dans les enquêtes épidémiologiques. Ce sont des grands facteurs qui permettent le regroupement des résultats en fonction de différentes catégories et qui sont des indicateurs de différents facteurs (comportementaux…) qui peuvent influer sur votre état de santé ou votre recours aux soins : âge, sexe, situation professionnelle, niveau d’étude.

These data include age, sex, professional situation, level of education and other information.

They are routinely collected in epidemiological surveys and are very important, as they both enable results to be grouped according to different categories, and may be indicators of different factors (behavioural, etc.) that can influence your state of health as well as your use of healthcare.

[**Votre expérience personnelle des événements**](https://impacts_epidemio.preprod.voozanoo.net/espa_13_novembre/)

**Your personal experience of the events**

Ces questions vont vous permettre d’exprimer objectivement et subjectivement ce que vous avez vécu et de quelle(s) façon(s) vous avez été impliqué(e) dans ces attentats.

These questions are designed to help you objectively and subjectively express what you experienced, and in what way(s) you were involved in these attacks.

Deux échelles psychométriques (Questionnaire des expériences de dissociation péritraumatique [PDEQ] et partie A3 du questionnaire Shortness of breath, tremulousness, racing heart and sweating rating scale [STRS]) vont également évaluer vos réactions émotionnelles aigues. Ces réactions peuvent influer sur votre état psychologique.

Two psychometric scales, i) the Peritraumatic Dissociation Experiences Questionnaire [PDEQ] and ii) part A3 of the Shortness of breath, tremulousness, racing heart and sweating rating scale [STRS] questionnaire), will also assess your acute emotional responses, as these types of responses can affect your psychological state.

[**Vos blessures physiques et leurs conséquences**](https://impacts_epidemio.preprod.voozanoo.net/espa_13_novembre/)

**Your physical injuries and their consequences**

Ces questions vont vous permettre d’exprimer les difficultés rencontrées du fait de vos blessures physiques. Il est important de pouvoir les prendre en compte pour construire une prise en charge globale associant une réparation somatique, psychique et sociale des personnes blessées physiquement et psychotraumatisées.

These questions will help you to express the difficulties you encountered as a result of your si si attack-related physical injuries, if you have any. It is important to take them into account in order to ensure comprehensive care which combines physical, psychological and social repair for people physically injured who have psychotrauma.

[**Concernant vos proches**](https://impacts_epidemio.preprod.voozanoo.net/espa_13_novembre/)

**Your family, friends, colleagues and acquaintances**

Cette partie aborde différents aspects que vous avez eu à affronter lors de la perte d’un proche dans ces attentats : annonce du décès, recherche de votre ou vos proches, constats du décès. Ces informations sont utiles pour aider à un meilleur accompagnement des personnes endeuillées.

This part of the study questionnaire looks at various aspects that people who lost one or more family members, friends, colleagues and acquaintances during the attacks have had to face: the announcement that someone died, the search for someone that died, death certificate(s), etc. This information is very useful to help provide better support to a bereaved person.

[**Ce que vous éprouvez psychologiquement**](https://impacts_epidemio.preprod.voozanoo.net/espa_13_novembre/)

**What you are experiencing psychologically**

Quatre échelles explorant les retentissements psychologiques pouvant se développer chez certaines personnes exposées à des événements traumatisants vont vous être posés. Ces questionnaires abordent l’état de stress post-traumatique, les symptômes anxieux et dépressifs, le risque suicidaire et le deuil compliqué (si vous avez perdu un proche lors des attentats). L’analyse des réponses à ces questionnaires permettra d’évaluer l’adéquation entre les symptômes et les soins psychiques qui ont pu être reçus et ainsi de proposer des pistes d’amélioration de la prise en charge.

In this part of the questionnaire, you will be invited to answer questions from four international scales which explore the psychological repercussions that can develop in some people who have been exposed to traumatic events. These scales deal with i) post-traumatic stress disorder, ii) symptoms of anxiety and depression, iii) the risk of suicide, and iv) complicated grief disorder (for example, if you lost a loved one during the attacks). Analysis of your answers to these questions will help assess how appropriate any psychological care which you received has been for the symptoms reported. In turn, this information will be used to suggest ways to improve care.

[**Le soutien psychologique que vous avez reçu**](https://impacts_epidemio.preprod.voozanoo.net/espa_13_novembre/)

**The psychological support you have received**

Cette partie aborde le soutien et les soins psychologiques que vous avez pu recevoir dans les suites immédiates des attentats jusqu’à aujourd’hui. Ces informations sont indispensables afin de permettre d’améliorer la prise en charge proposée au décours d’événements traumatiques.

This section discusses the psychological support and care you may have received from immediately after the attacks until today. This is essential information to improve the care offered after traumatic events.

S'il vous est difficile de remplir le questionnaire du fait de blessures physiques, de difficultés avec le français ou avec l'outil informatique, ou si vous vous sentez mal en remplissant le questionnaire, vous pouvez appeler ce numéro afin de prendre contact avec un psychologue (du lundi au samedi de 10h à 22h) : **N° non surtaxé - Info Conseil : 09 70 14 99 60**

If you have difficulties filling in the questionnaire because of physical injuries, difficulties with the language or difficulty using a computer, or if you do not feel well while filling in the questionnaire, you can call this number to contact a psychologist (Monday to Saturday from 10 a.m. to 10 p.m.): ***Info Conseil*: 09 70 14 99 60 (this is not a premium rate number)**

# Votre situation actuelle

# Your current situation

## Sexe

## Sex

Homme/Femme

Man/Woman

## Année de naissance (aaaa)

## Year of birth (yyyy)

## situation matrimoniale

## Conjugal status

Marié(e), pacsé(e) ou en union libre

Célibataire

Divorcé(e)

Veuf / Veuve

Married, in a civil union or De facto union

Single

Divorced

Widow/Widower

## Situation professionnelle

## Professional situation

En activité professionnelle

Au chômage

Au foyer

Étudiant

Retraité

Professionally active

Unemployed

Homemaker

Student

Retired

## Selon vous, de quelle catégorie votre profession se rapproche-t-elle le plus ?

## In your opinion, which category below most closely matches your profession?

Agriculteurs exploitants

Artisans, commerçants, chefs d’entreprise

Employés, ouvriers

Sans activité professionnelle

Cadres, prof. intellectuelles sup.

Professions intermédiaires (technicien)

Autre

Farmer

Craft worker, trader, business leader

Employee, blue-collar worker

No professional activity

Executive, professor, senior intellectual

Intermediate profession (e.g., technician)

Other

## Niveau d'étude

## What is your educational level?

Certificat d'études primaires (6 ans)

Formation professionnelle (CAP, BEP...)

Brevet des collèges

Baccalauréat

Études supérieures

Pas de diplôme

Primary school certificate (6 years of schooling)

Professional/Vocational training (Certificate of vocational proficiency (CAP), Professional diploma (BEP), etc.)

Junior high-school diploma

High-school diploma

Third-level studies

No educational diploma

# Votre expérience personnelle des événements

# Your personal experience of the events

## Dans quel(s) événement(s) avez-vous été impliqué(e) (vous étiez sur le lieu même, vous étiez proche géographiquement et vous vous êtes senti(e) menacé(e)) ?

## 2.1 From the list of events/places below, indicate which event/place (s) you were physically at the scene of, or which you were geographically close to and you felt threatened

### Stade de France

2.1.1 Stade de France Stadium

Oui-Non

Yes-No

### Rues Bichat et Alibert : Le Petit Cambodge, Bar le Carillon

Oui-Non

Yes-No

### Rue de la Fontaine au Roi : La Casa Nostra et La Bonne Bière

Oui-Non

Yes-No

### Rue de Charonne : La Belle Equipe

Oui-Non

Yes-No

### Boulevard Voltaire : Le Comptoir Voltaire

Oui-Non

Yes-No

### Le Bataclan

### 2.1.5 The Bataclan theatre

Oui –Non

Yes-No

### Assaut du 18 novembre à Saint-Denis Police assault on November 18 in Saint Denis

Oui –Non

Yes-No

---------------

## Stade de France

## 2.2 Stade de France stadium

### Lorsque les explosions se sont produites, vous étiez

2.2.1 When the explosions occurred, you were

À l’intérieur du stade de France

À l’extérieur du stade de France

Inside Stade de France stadium

Outside Stade de France stadium

#### A l’intérieur du stade de France

*2.2.1.1 Inside Stade de France stadium*

##### Avez-vous pensé que vous ou l’un de vos proches couriez un grave danger immédiat ?

2.2.1.1.1 Did you feel that you or a family member/friend/colleague/acquaintance was in serious immediate danger?

Oui –Non

Yes-No

##### Pouvez-vous nous expliquer en quoi vous vous êtes senti menacé(e) ?

2.2.1.1.2 Can you explain to us in what way you felt threatened? *Empty field*

### A l’extérieur du Stade de France

2.2.2 Outside Stade de France stadium

#### Où étiez-vous lorsque les terroristes ont déclenché leur ceinture d’explosifs ?

*2.2.2.1 Where were you when the terrorists set off their explosives belt?*

A moins de 20 mètres des terroristes

Entre 20 et 50 m des terroristes

Plus loin et vous avez vu les explosions et/ou leur conséquences

Less than 20 metres from the terrorists

Between 20 and 50 metres from the terrorists

Further away, and you saw the explosions and/or their consequences

#### Avez-vous vu l’explosion ?

#### Did you see an explosion?

Oui- Non

Yes-No

#### Avez-vous senti l’odeur de la poudre ou une autre odeur particulière provenant des lieux de l’événement ?

#### 2.2.2.3 Did you smell powder or any other strange smell coming from the scene of the event?

Oui- Non

Yes-No

#### Avez-vous ressenti le souffle de l’explosion ?

*2.2.2.4 Did you feel the blast of the explosion?*

Oui- Non

Yes-No

#### Avez-vous vu du sang ?

*2.2.2.5 Did you see blood ?*

Oui- Non

Yes-No

#### Avez-vous vu des personnes inertes, blessées, mutilées, mortes ou des restes de corps humains

*2.2.2.6 Did you see motionless, injured, mutilated or dead people or bodily remains?*

Oui- Non

Yes-No

#### Avez-vous touché des personnes blessées ou mortes ?

*2.2.2.7 Did you touch any injured or dead person?*

Oui- Non

Yes-No

#### Avez-vous été blessé(e) ?

*2.2.2.8 Were you physically injured?*

Oui- Non

Yes-No

#### Avez-vous reçu des projections de sang ou de restes de corps humains ?

2.2.2.9 Were you splattered by blood or were you hit by bodily remains?

Oui- Non

Yes-No

#### Avez-vous eu peur d’être blessé ?

*2.2.2.10 Were you afraid of being injured?*

Oui- Non

Yes-No

#### Avez-vous eu peur de mourir ?

#### 2.2.2.11 Were you afraid of dying?

Oui- Non

Yes-No

#### Avez-vous pu apporter de l’aide ?

*2.2.2.12 Were you able to help someone?*

Oui- Non

Yes-No

#### Avez-vous éprouvé un sentiment ou eu une perception de la situation non listés ici ?

*2.2.2.13 Did you experience any feeling or have any perception of the situation which is not listed here?*

Oui- Non

Yes-No

#### Préciser ce sentiment ou perception :

*2.2.2.14 Can you specify what this feeling or perception was?*

*empty field*

## Rues Bichat et Alibert : Le Petit Cambodge, Bar le Carillon ; Rue de la Fontaine au Roi : La Casa Nostra et La Bonne Bière ;Rue de Charonne : La Belle Equipe

## 2.3 Rues Bichat et Alibert : Le Petit Cambodge, Bar le Carillon ; Rue de la Fontaine au Roi : La Casa Nostra et La Bonne Bière ;Rue de Charonne : La Belle Equipe

### Où étiez-vous lorsque les terroristes ont ouvert le feu ?

**2.3.1 Where were you when the terrorists opened fire?**

Dans luncafé visé ou à sa terrasse

Dans la rue à proximité des cafés visés

Dans une rue adjacente

In a targeted café or its terrace

In the same street as targeted cafes

In an adjacent street

#### SI dans la rue à proximité des cafés visé

*2.3.1.1 IF you were in an adjacent street to targeted cafes*

Etiez-vous

A pied

En voiture

Dans un bâtiment

Were you
On foot

In a car

In a building

### Avez-vous fui l’événement pour vous mettre à l’abri ?

2.3.2 Did you flee the event to take cover?

###

oui-non

Yes-No

### Avez-vous senti l’odeur de la poudre ou une autre odeur particulière provenant des lieux de l’événement ?

2.3.3 Did you smell powder or any other strange smell coming from the scene of the event?

oui-non

Yes-No

### Avez-vous vu du sang ?

2.3.4 Did you see blood ?

oui-non

Yes-No

### Avez-vous vu les terroristes ?

2.3.5 Did you see the terrorists?

oui-non

Yes-No

### Avez-vous croisé le regard des terroristes ?

2.3.6 Did you make eye contact with the terrorists?

oui-non

Yes-No

### Vous êtes-vous senti visé(e) par les tirs ?

2.3.7 Did you feel you were targeted by the shots?

oui-non

Yes-No

### Avez-vous vu directement une personne être menacée, blessée ou mourir ?

### 2.3.8 Did you directly see someone be threatened, get injured or die ?

oui-non

Yes-No

### Avez-vous vu des personnes inertes, blessées, mutilées, mortes ?

2.3.9 Did you see any motionless, injured, mutilated or dead people?

oui-non

Yes-No

### Avez-vous touché des personnes blessées mutilées ou mortes ?

### 2.3.10 Did you touch any injured, mutilated or dead person?

oui-non

Yes-No

### Avez-vous cru que vous alliez mourir ?

### 2.3.11 Did you think you were going to die?

oui-non

Yes-No

### Avez-vous été blessé(e) ?

2.3.12 Were you injured ?

oui-non

Yes-No

### Vous êtes vous retrouvé(e) dans l’impossibilité d’apporter de l’aide ?

2.2.13 Did you find yourself unable to provide help?

oui-non

Yes-No

### Avez-vous éprouvé un sentiment ou eu une perception de la situation non listés ici ?

2.3.14 Did you experience any feeling or have any perception of the situation which is not listed here?

oui-non

Yes-No

### Avez-vous pu aussi apporter de l’aide ?

2.3.15 Were you able to help someone?

oui-non

Yes-No

### Préciser ce sentiment / perception

2.3.16 Can you specify what this feeling or perception was?

*empty field*

## Boulevard Voltaire

## 2.4 Boulevard Voltaire

### Où étiez-vous lorsque le terroriste a déclenché sa ceinture d’explosifs ?

2.4.1 Where were you when the terrorist detonated his explosive belt?

Dans le café visé ou à sa terrasse

Dans la rue à proximité des cafés visés

In the targeted café or its terrace

In the same street as the targeted cafes

- Etiez-vous ?
- A pied
- En voiture
- Dans un bâtiment

Dans une rue adjacente

Were you ?

- On foot
- In a car
- In a building
- In an adjacent street

#### Si dans la rue à proximité

*2.4.1.1 IF you were in an adjacent street*

### Avez-vous vu le terroriste ?

2.4.2 Did you see the terrorists?

oui-non

Yes-No

### Avez-vous vu l’explosion ?

2.4.3 Did you see the explosion?

oui-non

Yes-No

### Avez-vous senti l’odeur de la poudre ou une autre odeur particulière provenant des lieux de l’événement

#### 2.4.4. Did you smell powder or any other strange smell coming from the scene of the event?

oui-non

Yes-No

### Avez-vous ressenti le souffle de l’explosion ?

2.4.5 Did you feel the blast of the explosion?

oui-non

Yes-No

### Avez-vous vu du sang ?

2.4.6 Did you see blood ?

oui-non

Yes-No

### Avez-vous vu des personnes inertes, blessées, mutilées, mortes ou des restes de corps humains ?

2.4.7 Did you see any motionless, injured, mutilated or dead people or bodily remains?

oui-non

Yes-No

### Avez-vous touché des personnes blessées, mutilées ou mortes ?

### 2.4.8 Did you touch any injured, mutilated or dead person?

oui-non

Yes-No

### Avez-vous reçu des projections de sang ou de restes de corps humains ?

### 2.4.9 Were you splattered by blood or were you hit by bodily remains?

oui-non

Yes-No

### Avez-vous été blessé(e) ?

2.4.10 Were you phyically injured ?

oui-non

Yes-No

### Si Avez-vous cru que vous alliez mourir ?

2.4.11 Did you think you were going to die?

oui-non

Yes-No

### Avez-vous pu aussi apporter de l’aide ?

2.4.12 Were you able to help someone?

oui-non

Yes-No

#### Quel type d'aide ?

### Avez-vous éprouvé un sentiment ou eu une perception de la situation non listés ici ?

2.4.13 Did you experience any feeling or have any perception of the situation which is not listed here?

oui-non

Yes-No

#### Préciser ce sentiment / perception

*2.4.13.1 Can you specify what this feeling or perception was?*

#### empty field

## Bataclan

2.5 The Bataclan theatre

### Vous étiez :

2.5.1 You were :

A l'intérieur du Bataclan

A proximité immédiate du bataclan

Inside the Bataclan theatre

In the immediate vicinity of the Bataclan theatre

### Si vous étiez dans le bataclan

2.5.2 If you were inside the Bataclan theatre

#### Avez-vous pu vous enfuir à l'extérieur du Bataclan ?

*2.5.2.1 Were you able to flee to the outside of the theatre?*

Oui/non

Yes

no

##### Etait-ce :

2.5.2.1.1 Did you flee:?

Par le toit

Par une issue de secours

Autre

Through the roof

Through an emergency exit

Other

###### Précisez

2.5.2.1.1.1 Specify

#### Etiez-vous dans la fosse ?

*2.5.2.2 Were you in the orchestra pit?*

Oui-Non

Yes-No

#### Avez-vous pu vous cacher à l'intérieur du Bataclan ?

2.5.2.2 Were you able to hide inside the Bataclan theatre?

Oui-Non

Yes-No

##### Etait-ce :

2.5.2.3.1 Did you hide ?:

Dans un faux plafond

Dans les toilettes

Dans un local technique

Dans la loge

Dans les bureaux

Dans une boutique de tatouage

Sous le bar

Dans les coulisses

Autre

In a false ceiling

In the toilets

In a control room

In the artists’ dressing room

In offices

In the tattoo shop located in the theatre

Under the bar

In the wings

Other

##### Précisez

2.5.2.3.2 Specify

#### Avez-vous senti l’odeur de la poudre ou une autre odeur particulière provenant des lieux de l’événement ?

*2.5.2.4 Did you smell powder or any other strange smell coming from the scene of the event?*

oui-non

Yes-No

#### Avez-vous vu du sang ?

*2.5.2.5 Did you see blood ?*

oui-non

Yes-No

#### Avez-vous vu des personnes inertes, blessées, mutilées ou mortes ?

*2.5.2.6 Did you see any motionless, injured, mutilated or dead people?*

oui-non

Yes-No

#### Avez-vous touché des personnes blessées ou mortes ?

*2.5.2.7 Did you touch any injured,or dead person?*

oui-non

Yes-No

#### Avez-vous vu directement quelqu’un être menacé, blessé ou mourir ?

*2.5.2.8 Did you directly see someone be threatened, get injured or die?*

oui-non

Yes-No

#### Avez-vous entendu des personnes mourir ?

*2.5.2.9 Did you hear people die?*

oui-non

Yes-No

#### Avez-vous croisé le regard des terroristes ?

*2.5.2.10 Did you make eye contact with the terrorists?*

oui-non

Yes-No

#### Avez-vous vu directement les terroristes ?

*2.5.2.11 Did you directly see the terrorists?*

oui-non

Yes-No

#### Avez-vous perçu la présence des terroristes à proximité de vous ?

*2.5.2.12 Did you feel the presence of the terrorists close to you?*

oui-non

Yes-No

#### Avez-vous dû parler avec les terroristes ?

*2.5.2.13 Did you have to speak with the terrorists?*

oui-non

Yes-No

#### Avez-vous été visé par les terroristes ?

*2.5.2.14 Were you targeted by the terrorists?*

oui-non

Yes-No

#### Un terroriste vous a-t-il physiquement touché ?

#### 2.5.2.15 Did a terrorist physically touch you ?

oui-non

Yes-No

#### Avez-vous simulé votre mort ?

*2.5.2.16 Did you pretend to be dead?*

oui-non

Yes-No

#### Vous êtes-vous protégé à l’aide de corps ?

#### 2.5.2.17 Did you protect yourself using a body (ies)?

oui-non

Yes-No

#### Avez-vous eu peur qu’un bruit dénonce votre présence (sonnerie de téléphone, cri, pleurs etc.) ?

#### 2.5.2.18 Were you afraid that a noise would give away your presence (phone ringing, screaming, crying, etc.)?

oui-non

Yes-No

#### Vous êtes-vous retrouvé(e) dans l’impossibilité d’apporter de l’aide ?

2.5.2.19 Did you find yourself unable to provide help?

oui-non

Yes-No

#### Avez-vous pu aussi apporter de l’aide ?

*2.5.2.20 Were you able to help someone?*

oui-non

Yes-No

##### Préciser le type d'aide

2.5.2.20.1 Specify what type of help you were able to give

#### Avez-vous eu connaissance que la police allait intervenir ?

*2.5.2.21 Did you know that the police were going to intervene?*

oui-non

Yes-No

#### Avez-vous éprouvé un sentiment ou eu une perception de la situation non listés ici ?

*2.5.2.21 Did you experience any feeling or have any perception of the situation which is not listed here?*

oui-non

Yes-No

#### Préciser ce sentiment / perception :

*2.5.2.23 Can you specify what this feeling or perception was?*

### Bataclan extérieur (proximité immédiate)

2.5.3 Outside the Bataclan theatre (in the immediate vicinity)

#### Avez-vous été confiné ou dû vous mettre à l’abri?

*2.5.3.1 Were you in a confined space (e.g., false ceiling or wardrobe) or had you to take cover?*

oui-non

Yes-No

#### Avez-vous senti l’odeur de la poudre ou une autre odeur particulière provenant des lieux de l’événement ?

*2.5.3.2 Did you smell powder or any other strange smell coming from the scene of the event?*

oui-non

Yes-No

#### Avez-vous vu du sang ?

*2.5.3.3 Did you see blood ?*

oui-non

Yes-No

#### Avez-vous vu des personnes inertes, blessées, mutilées, mortes ou des restes de corps humains ?

*2.5.3.4 Did you see any motionless, injured, mutilated or dead people or bodily remains?*

oui-non

Yes-No

#### Avez-vous touché des personnes blessées ou mortes ?

2.5.3.5 *Did you touch any injured, or dead person?*

oui-non

Yes-No

#### Avez-vous vu directement quelqu’un être menacé, blessé ou mourir ?

*2.5.3.6 Did you directly see someone be threatened, get injured or die ?*

oui-non

Yes-No

#### Avez-vous éprouvé un sentiment ou eu une perception de la situation non listés ici ?

2.5.3.7 Did you experience any feeling or have any perception of the situation which is not listed here?

Oui- Non

Yes-No

##### Laquelle

2.5.3.7.1 Can you specify what this feeling or perception was?

#### Avez-vous pu apporter de l’aide ?

*2.5.3.8 Were you able to help someone?*

Oui_Non

Yes-No

##### Laquelle

2.5.3.8.1 Specify what type of help you were able to give

## Assaut du 18 Novembre

2.6 Police assault on November 18th in Saint Denis

### Où étiez-vous lors de l'assaut du 18 novembre à Saint-Denis ?

2.6.1 Where were you during the police assault on November 18^th^ in Saint Denis ?

Dans l'immeuble du 48 rue de la République

Dans un immeuble proche

In the building at 48 Rue de la République

In a nearby building

### Avez-vous été confiné pendant l'assaut ?

2.6.2 Had you to take cover during the assault?

oui-non

Yes-No

### Votre logement a-t-il été endommagé par des tirs ou par l'explosion ?

2.6.3 Was the place in you were living damaged by bullets or the explosion?

oui-non

Yes-No

### Votre logement est –il devenu inhabitable suite à cet assaut ?

### 2.6.4 Did the place in you were living become uninhabitable after the assault?

oui-non

Yes-No

### Avez-vous vu du sang ?

2.6.5 Did you see blood ?

oui-non

Yes-No

### Avez-vous vu des personnes inertes, blessées, mutilées, mortes ou des restes de corps humains ?

2.6.6 Did you see motionless, injured, mutilated or dead people or bodily remains?

oui-non

Yes-No

### Avez-vous senti l’odeur de la poudre ou une autre odeur particulière provenant des lieux de l’événement ?

2.6.7 Did you smell powder or any other strange smell coming from the scene of the event?

oui-non

Yes-No

### Avez-vous été blessé(e) ?

2.6.8 Were you injured ?

oui-non

Yes-No

### Avez-vous reçu des projections de sang ou de restes de corps humains ?

2.6.9 Were you splattered by blood or were you hit by bodily remains?

oui-non

Yes-No

### Avez-vous ressenti le souffle de l’explosion ?

### 2.6.10 Did you feel the blast of the explosion?

oui-non

Yes-No

### Avez-vous éprouvé un sentiment ou eu une perception de la situation non listés ici ?

2.6.11 Did you experience any feeling or have any perception of the situation which is not listed here?

oui-non

Yes-No

### Laquelle

2.6.12 Can you specify what this feeling or perception was?

### Si vous ne vous êtes pas reconnu(e) dans les questions précédentes, pouvez-vous exprimer en quoi vous vous êtes senti exposé(e) (bouleversé(e)) ?

**2.6.13 If you did not recognize yourself in the previous questions, can you express how you felt exposed (upset)?**

# Vos blessures physiques et leurs conséquences

# 3 Your physical injuries and their consequences

## Avez-vous été blessé(e) physiquement lors des attentats ?

## 3.1 Were you physically injured during the attacks?

Oui-Non

Yes-No

## Vos soins ont nécessité une hospitalisation initiale (en urgence) qui s’est prolongée plus d’une semaine

## 3.2 Did the care which you initially received require emergency hospitalization that lasted for more than one week?

Oui- Non

Yes-No

### Combien de semaines ?

3.2.1 How many weeks ?

## Vos soins ont nécessité une hospitalisation initiale (en urgence) de moins d’une semaine

## 3.3 Did the care which you initially received require emergency hospitalization that lasted for less than one week?

Oui-Non

Yes-No

## Vos soins ont nécessité une hospitalisation programmée/différée

## 3.4 Did the care you received require planned/deferred hospitalization?

Oui-Non

Yes-No

## Et maintenant pourriez-vous nous informer sur ce qui s’est passé pendant l’hospitalisation (première hospitalisation si plusieurs) pour votre ou vos blessures physiques

## In the following questions we will focus on what happened during hospitalization (or your first hospitalization if you were hospitalised more than once) for your physical injury(s)

## Avez-vous bénéficié d’un soutien psychologique par un psychiatre ou un psychologue pendant l’hospitalisation ?

## 3.5 Did you receive psychological support from a psychiatrist or psychologist during hospitalization?

Oui-Non

Yes-No

# Concernant vos proches

# 4 Your family/ friends/ colleagues/acquaintances

## Avez-vous un ou plusieurs proches qui ont été directement menacés par les terroristes et/ou blessés physiquement ou psychiquement lors de ces attentats

## 4.1 Do you have one or more relatives/friends/colleagues/acquaintances who were directly threatened by the terrorists and /or physically or mentally injured during these attacks?

Oui-Non

Yes-No

### Étai(en)t-ce

4.1.1 Is this relative/friend/colleague/acquaintance

Enfant

Un(e) conjoint(e)

Grand parent

Un arrière grand parent

Un petit enfant

Un arrière petit enfant

Un parent

Un autre membre de votre famille

Un ami

Un(e) collègue

Une connaissance

Autre

Étai(en)t-il(s)

Avec vous

Sur un autre lieu d'attentat

Your own child

A spouse

A grandparent

A great grandparent

A grandchild

A great grandchild

A parent

Another member of your family

A friend

A colleague

An acquaintance

Other

Was he/she/they:

With you

At the scene of another attack

Si autre préciser

If so, specify the location of the other attack

## Avez-vous perdu un ou plusieurs proches lors de ces attentats ?

## 4.2 Did you lose one or more relative/friend/colleague/acquaintances during these attacks?

Oui-Non

Yes-No

### Quel était votre lien avec la ou les personnes décédées dans les attentats ? (plusieurs réponses possibles)

4.2.1 What was your relationship to your relative/friend/colleague/acquaintance(s) who died in the attacks? (one answer for each deceased person)

Enfant

Un(e) conjoint(e)

Grand parent

Un arrière grand parent

Un petit enfant

Un arrière petit enfant

Un parent

Un autre membre de votre famille

Un ami

Un(e) collègue

Une connaissance

Autre

Si autre préciser

Your own child

A spouse

A grandparent

A great grandparent

A grandchild

A great grandchild

A parent

Another member of your family

A friend

A colleague

An acquaintance

Other

If other, specify

### Etai(en)t-il(s) avec vous au moment où ils sont morts ? (plusieurs réponses possibles)

4.2.2. Were they with you when he/she/they died?

Oui-Non

Yes-No

# Ce que vous éprouvez psychologiquement

# 5 What you are experiencing psychologically

## The Posttraumatic Stress Disorder Checklist (PCL-5)

## 5.1 The Post-traumatic Stress Disorder Checklist (PCL-5)

Voici une liste de problèmes que les gens éprouvent parfois suite à une expérience vraiment stressante (ici il est question de ces attentats et de votre vécu de ces attentats). Veuillez lire chaque énoncé attentivement et cocher pour indiquer dans quelle mesure ce problème vous a affecté dans le dernier mois.

Below is a list of problems that people sometimes have in response to a very stressful experience (in our case, this stressful experience regards the November 15 attacks/November 18 police assault, and your experience of these events). Please read each statement carefully and tick to indicate how this issue has affected you in the past month.

Dans le dernier mois, dans quelle mesure avez-vous été affecté par

In the past month, how much were you bothered by:

Pas du tout-Un peu-Moyennement-Souvent-Extrêmement

### Des souvenirs répétés, pénibles et involontaires de l’expérience stressante ? [ champ_lpcl5_1 ]

### 5.1.1 Repeated, disturbing, and unwanted memories of the stressful experience? [PCL-5 item 1]

Pas du tout-Un peu-Moyennement-Souvent-Extrêmement

Not at all A little bit Moderately Quite a bit Extremely

### Des rêves répétés et pénibles de l’expérience stressante? [ champ_lpcl5_2 ]

### 5.1.2 Repeated, disturbing dreams of the stressful experience? [PCL-5 item 2]

Pas du tout-Un peu-Moyennement-Souvent-Extrêmement

Not at all A little bit Moderately Quite a bit Extremely

### Se sentir soudainement comme si l’expérience stressante recommençait (comme si vous la viviez de nouveau)? [ champ_lpcl5_3 ]

### 5.1.3 Suddenly feeling or acting as if the stressful experience were actually happening again (as if you were actually back there reliving it)? [PCL-5 item 3]

Pas du tout-Un peu-Moyennement-Souvent-Extrêmement

Not at all A little bit Moderately Quite a bit Extremely

### Être bouleversé(e) lorsque quelque chose vous rappelle l’expérience stressante? [ champ_lpcl5_4 ]

### 5.1.4 Feeling very upset when something reminded you of the stressful experience? [PCL-5 item 4]

###

Pas du tout-Un peu-Moyennement-Souvent-Extrêmement

Not at all A little bit Moderately Quite a bit Extremely

### Réagir physiquement lorsque quelque chose vous rappelle l’expérience stressante (p. ex., avoir le coeur qui bat très fort, du mal à respirer, ou avoir des sueurs)? [ champ_lpcl5_5

### 5.1.5 Having strong physical reactions when something reminded you of the stressful experience (for example, heart pounding, trouble breathing, sweating)? [PCL-5 item 5]

Pas du tout-Un peu-Moyennement-Souvent-Extrêmement

Not at all A little bit Moderately Quite a bit Extremely

### Éviter souvenirs, pensées ou sentiments en lien avec l’expérience stressante? [ champ_lpcl5_6 ]

### 5.1.6 Avoiding memories, thoughts, or feelings related to the stressful experience? [PCL-5 item 6]

Pas du tout-Un peu-Moyennement-Souvent-Extrêmement

Not at all A little bit Moderately Quite a bit Extremely

### Éviter les personnes et les choses qui vous rappellent l’expérience stressante (p. ex., des gens, des lieux, des conversations, des activités, des objets, ou des situations)? [ champ_lpcl5_7 ]

### 5.1.7 Avoiding external reminders of the stressful experience (for example, people, places, conversations, activities, objects, or situations)? [PCL-5 item 7]

Pas du tout-Un peu-Moyennement-Souvent-Extrêmement

Not at all A little bit Moderately Quite a bit Extremely

### Avoir du mal à vous rappeler d’éléments importants de l’expérience stressante? [ champ_lpcl5_8 ]

### 5.1.8 Trouble remembering important parts of the stressful experience? [PCL-5 item 8]

Pas du tout-Un peu-Moyennement-Souvent-Extrêmement

Not at all A little bit Moderately Quite a bit Extremely

### Avoir des croyances négatives sur vous-même, les autres ou sur le monde (p. ex., avoir des pensées telles que : je suis mauvais, il y a quelque chose qui cloche sérieusement chez moi, nul n’est digne de confiance, le monde est un endroit complètement dangereux)? [ champ_lpcl5_9 ]

### 5.1.9 Having strong negative beliefs about yourself, other people, or the world (for example, having thoughts such as: I am bad, there is something seriously wrong with me, no one can be trusted, the world is completely dangerous)? [PCL-5 item 9]

Pas du tout-Un peu-Moyennement-Souvent-Extrêmement

Not at all A little bit Moderately Quite a bit Extremely

### Vous blâmer ou blâmer les autres pour la survenue de l’expérience stressante ou ce qui est arrivé par la suite? [ champ_lpcl5_10 ]

### 5.1.10 Blaming yourself or someone else for the stressful experience or what happened after it? [PCL-5 item 10]

Pas du tout-Un peu-Moyennement-Souvent-Extrêmement

Not at all A little bit Moderately Quite a bit Extremely

### Avoir des sentiments négatifs intenses tels que peur, horreur, colère, culpabilité, ou honte? [ champ_lpcl5_11 ]

### 5.1.11 Having strong negative feelings such as fear, horror, anger, guilt, or shame? [PCL-5 item 11]

Pas du tout-Un peu-Moyennement-Souvent-Extrêmement

Not at all A little bit Moderately Quite a bit Extremely

### Perdre de l’intérêt pour des activités que vous aimiez auparavant? [ champ_lpcl5_1

### 5.1.12 Loss of interest in activities that you used to enjoy? [PCL-5 item 12]

Pas du tout-Un peu-Moyennement-Souvent-Extrêmement

Not at all A little bit Moderately Quite a bit Extremely

### Vous sentir distant ou coupé des autres? [ champ_lpcl5_13 ]

### 5.1.13 Feeling distant or cut off from other people? [PCL-5 item 13]

Pas du tout-Un peu-Moyennement-Souvent-Extrêmement

Not at all A little bit Moderately Quite a bit Extremely

### Avoir du mal à éprouver des sentiments positifs (p. ex., être incapable de ressentir la joie ou de l’amour envers vos proches)? [ champ_lpcl5_14 ]

### 5.1.14 Trouble experiencing positive feelings (for example, being unable to feel happiness or have loving feelings for people close to you)? [PCL-5 item 14]

Pas du tout-Un peu-Moyennement-Souvent-Extrêmement

Not at all A little bit Moderately Quite a bit Extremely

### Être irritable, avoir des bouffées de colère, ou agir agressivement? [ champ_lpcl5_15 ] 5.1.15 Irritable behaviour, angry outbursts, or acting aggressively? [PCL-5 item 15]

Pas du tout-Un peu-Moyennement-Souvent-Extrêmement

Not at all A little bit Moderately Quite a bit Extremely

### Prendre des risques inconsidérés ou encore avoir des conduites qui pourraient vous mettre en danger ? [ champ_lpcl5_16 ]

### 5.1.16 Taking too many risks or doing things that could cause you harm? [PCL-5 item 16]

Pas du tout-Un peu-Moyennement-Souvent-Extrêmement

Not at all A little bit Moderately Quite a bit Extremely

### Être en état de ‘super-alerte’, vigilant ou sur vos gardes? [ champ_lpcl5_17 ]

### 5.1.17 Being “superalert” or watchful or on guard? [PCL-5 item 17]

Pas du tout-Un peu-Moyennement-Souvent-Extrêmement

Not at all A little bit Moderately Quite a bit Extremely

### Sursauter facilement? [ champ_lpcl5_18 ]

### 5.1.18 Feeling jumpy or easily startled? [PCL-5 item 18]

Pas du tout-Un peu-Moyennement-Souvent-Extrêmement

Not at all A little bit Moderately Quite a bit Extremely

### Avoir des difficultés de concentration [champ_lpc5_19]]

### 5.1.19 Having difficulty concentrating ? [PCL-5 item 19]

Pas du tout-Un peu-Moyennement-Souvent-Extrêmement

Not at all A little bit Moderately Quite a bit Extremely

### Avoir du mal à trouver ou garder le sommeil? [ champ_lpcl5_20 ]

### 5.1.20 Trouble falling or staying asleep? [PCL-5 item 20 ]

Pas du tout-Un peu-Moyennement-Souvent-Extrêmement

Not at all A little bit Moderately Quite a bit Extremely

### Est-ce que ces difficultés rendent vos relations avec votre famille plus difficiles ?

5.1.21 Do these problems make your relationships with your family members more difficult?

Oui-Non

Yes-No

### Est-ce que ces difficultés vous posent des problèmes pour vous entendre avec vos amis ?

5.1.22 Do these problems make it difficult for you to get on with friends?

Oui-Non

Yes-No

### Est-ce que ces difficultés vous posent des problèmes pour bien travailler ?

5.1.23 Do these problems make it difficult for you to work effectively?

Oui-Non

Yes-No

### Est-ce que ces difficultés vous posent des problèmes pour votre niveau général de fonctionnement dans la vie ?

5.1.24 Do these problems make it difficult for your daily general functioning?

Oui-Non

Yes-No

## Echelle HAD : Hospital anxiety and depression scale

## 5.2 Echelle HAD : Hospital anxiety and depression scale

Instructions : ce questionnaire a été conçu de façon à vous permettre d’exprimer ce que vous éprouvez sur le plan émotif. Lisez chaque question et choisissez la réponse qui exprime le mieux ce que vous avez éprouvé au cours des 7 jours qui viennent de s’écouler.

Ne vous attardez pas sur la réponse à faire : votre réaction immédiate à chaque question fournira probablement une meilleure indication de ce que vous éprouvez qu’une réponse longuement méditée.

Instructions: This questionnaire was designed in order to allow you to express how you are feeling emotionally. Read each question below and choose the answer that best expresses what you have felt during the past 7 days.

Don't take too long over your replies: your immediate reaction to each question will likely provide a better indication of what you are feeling than a long thought-out answer.

DANS LES 7 DERNIERS JOURS QUI VIENNENT DE S'ECOULER

IN THE PAST 7 DAYS

### Je me sens tendu(e) ou énervé(e)

### 5.2.1 I feel tense or 'wound up'

La plupart du temps

Souvent

De temps en temps

Jamais

Most of the time

A lot of the time

From time to time, occasionally

Not at all

### Je prends plaisir aux mêmes choses qu’autrefois

### 5.2.2 I still enjoy the things I used to enjoy

Oui, tout autant qu’avant

Pas autant

Un peu seulement

Presque plus

Definitely as much

Not quite so much

Only a little

Hardly at all

### J’ai une sensation de peur comme si quelque chose d’horrible allait m’arriver

### 5.2.3 I get a sort of frightened feeling as if something awful is about to happen

Oui, très nettement

Oui, mais ce n’est pas trop grave

Un peu, mais cela ne m’inquiète pas

Pas du tout

Very definitely and quite badly

Yes, but not too badly

A little, but it doesn't worry me

Not at all

### Je ris facilement et vois le bon côté des choses

### 5.2.4 I can laugh and see the funny side of things

Autant que par le passé

Plus autant qu’avant

Vraiment moins qu’avant

Plus du tout

As much as I always could

Not quite so much now

Definitely not so much now

Not at all

### Je me fais du souci

### 5.2.5 Worrying thoughts go through my mind

Très souvent

Assez souvent

Occasionnellement

Très occasionnellement

A great deal of the time

A lot of the time

From time to time but not too often

Only occasionally

### Je suis de bonne humeur

### 5.2.6 I feel cheerful

Jamais

Rarement

Assez souvent

La plupart du temps

Not at all

Not often

Sometimes

Most of the time

### Je peux rester tranquillement assis(e) à ne rien faire et me sentir décontracté(e)

### 5.2.7 I can sit at ease and feel relaxed

Oui, quoi qu’il arrive

Oui, en général

Rarement

Jamais

Definitely

Usually

Not often

Not at all

### J’ai l’impression de fonctionner au ralenti

### 5.2.8 I feel as if I am slowed down

Presque toujours

Très souvent

Parfois

Jamais

Nearly all the time

Very often

Sometimes

Not at all

### J’éprouve des sensations de peur et j’ai l’estomac noué

### 5.2.9 I get a sort of frightened feeling like 'butterflies' in the stomach

Jamais

Parfois

Assez souvent

Très souvent

Not at all

Occasionally

Quite often

Very often

### Je ne m’intéresse plus à mon apparence

### 5.2.10 I have lost interest in my appearance

Plus du tout

Je n’y accorde pas autant d’attention que je devrais

Il se peut que je n’y fasse plus autant attention

J’y prête autant d’attention que par le passé

Definitely

I don't take so much care as I should

I may not take quite as much care

I take just as much care as ever

### J’ai la bougeotte et n’arrive pas à tenir en place

### 5.2.11 I feel restless as if I have to be on the move

Oui, c’est tout à fait le cas

Un peu

Pas tellement

Pas du tout

Very much indeed

Quite a lot

Not very much

Not at all

### Je me réjouis d’avance à l’idée de faire certaines choses

### 5.2.12 I look forward with enjoyment to things

Autant qu’avant

Un peu moins qu’avant

Bien moins qu’avant

Presque jamais

As much as ever I did

Rather less than I used to

Definitely less than I used to

Hardly at all

### J’éprouve des sensations soudaines de panique

### 5.2.13 I get sudden feelings of panic:

Vraiment très souvent

Assez souvent

Pas très souvent

Jamais

Very often indeed

Quite often

Not very often

Not at all

### Je peux prendre plaisir à un bon livre ou à une bonne émission de radio ou de télévision

### 5.2.14 I can enjoy a good book or radio or TV programme

Souvent

Parfois

Rarement

Très rarement

Often

Sometimes

Not often

Very seldom

# Le soutien psychologique que vous avez reçu

# 6 The psychological support you have received

## Avant d’avoir pu regagner votre lieu d’hébergement

## 6.1 Before being able to return to your living accommodation

### Avez-vous des souvenirs de ce que vous avez vécu entre les suites immédiates de l'événement et le moment où vous avez regagné votre lieu d'hébergement ?

6.1.1 Do you have any memories of what you experienced between the immediate aftermath of the event and the moment when you returned to your living accommodation?

Oui –Non

Yes-No

### Avez-vous le souvenir d’avoir reçu un soutien ou une aide ?

6.1.2 Do you remember receiving any support or assistance?

Oui-Non

Yes-No

### Cette aide ou ce soutien étaient apportés par

6.1.3 Who provided this support or assistance?

Personnel non identifié

pompiers

SAMU

cump

Autre personne santé

Non-identified personnel

Firefighters

Emergency medical services (SAMU)

Emergency outreach psychosocial support unit (CUMP)

Other healthcare providers

#### Des personnels non identifiés

6.1.3.1 Non-identified personnel

##### Pourriez-vous dire dans quel(s) lieu(x)?

6.1.2.1.1 Can you tell us in which place(s)?

Sur le lieu de l’événement

Dans la rue

A l’Hôtel Dieu

Dans un autre hôpital

Dans une mairie

Dans une école

Au commissariat

Ailleurs

Je ne sais pas

At the scene of the event

In the street

At Hôtel Dieu hospital

In another hospital

In a town hall

In a school

At a police station

Elsewhere

I do not know

###### Si ailleurs préciser

6.1.3.1.1.1 If elsewhere, specify the place(s)

#### Pompiers

6.1.3.2 Firefighters

##### Pourriez-vous dire dans quel(s) lieu(x)?

6.1.3.2.1 Can you tell us in which place(s)?

Sur le lieu de l’événement

Dans la rue

A l’Hôtel Dieu

Dans un autre hôpital

Dans une mairie

Dans une école

Au commissariat

Ailleurs

Je ne sais pas

At the scene of the event

In the street

At Hôtel Dieu hospital

In another hospital

In a town hall

In a school

At a police station

Elsewhere

I do not know

###### si ailleurs préciser

6.1.3.2.1.1 If elsewhere, specify the place(s)

#### SAMU

*6.1.3.3 Emergency medical services (SAMU)*

##### Pourriez-vous dire dans quel(s) lieu(x)?

6.1.3.3.1 Can you tell us in which place(s)?

Sur le lieu de l’événement

Dans la rue

A l’Hôtel Dieu

Dans un autre hôpital

Dans une mairie

Dans une école

Au commissariat

Ailleurs

Je ne sais pas

At the scene of the event

In the street

At Hôtel Dieu hospital

In another hospital

In a town hall

In a school

At a police station

Elsewhere

I do not know

###### si ailleurs préciser

6.1.3.3.1.1 If elsewhere, specify the place(s)

#### CUMP

*6.1.3.4 Emergency outreach psychosocial support unit (CUMP)*

##### Pourriez-vous dire dans quel(s) lieu(x)?

6.1.3.4.1 Can you tell us in which place(s)?

Sur le lieu de l’événement

Dans la rue

A l’Hôtel Dieu

Dans un autre hôpital

Dans une mairie

Dans une école

Au commissariat

Ailleurs

Je ne sais pas

At the scene of the event

In the street

At Hôtel Dieu hospital

In another hospital

In a town hall

In a school

At a police station

Elsewhere

I do not know

###### Si ailleurs préciser

6.1.3.4.1.1 If elsewhere, specify the place(s)

#### Autre personnel de soin

*6.1.3.5 Other healthcare providers*

##### Pourriez-vous dire dans quel(s) lieu(x)?

6.1.3.5.1 Can you tell us in which place(s)?

Sur le lieu de l’événement

Dans la rue

A l’Hôtel Dieu

Dans un autre hôpital

Dans une mairie

Dans une école

Au commissariat

Ailleurs

Je ne sais pas

At the scene of the event

In the street

At Hôtel Dieu hospital

In another hospital

In a town hall

In a school

At a police station

Elsewhere

I do not know

###### si ailleurs préciser

6.1.3.5.1.1 If elsewhere, specify the place(s)

## Avant d’avoir pu regagner votre lieu d’hébergement, avez-vous été hospitalisé(e) pour des raisons psychologiques ?

## 6.2 Before being able to return to your living accommodation, were you hospitalized for psychological reasons?

Oui – Non

Yes-No

## Depuis les événements (ou après avoir regagné votre lieu d’hébergement pour les personnes sur les lieux ou à proximité au moment des attaques) Êtes-vous allé(e) dans un de ces lieux d’accueil mis en place juste après les attentats ?

## 6.3 After the events (or after returning to your living accommodation for people on or close to the scene at the time of the attacks/assault), did you go to any of the reception centres which were set up in the days following the attacks?

Mairie du 10e/école Parmentier

Mairie du 11e

Mairie de Saint-Denis ou centre municipal de santé rue du Cygne

Institut médico-légal

Ecole Militaire

Hôtel-Dieu

Autre

10^th^ district town hall or adjacent Parmentier school

11^th^ district town hall

Saint-Denis town hall or Municipal healthcare centre located in Rue du Cygne

Institute of Forensic Medicine

National Military school

Hotel-Dieu hospital

Other

#### Si autre préciser

*6.3.1.1 If elsewhere, specify the place(s)*

### Mairie du 10e/école Parmentier

6.3.2 10^th^ district town hall or Parmentier school

#### 6.3.2.1 Avez-vous bénéficié d’un soutien psychosocial des professionnels de santé (CUMP, service de santé des armées, etc) ?

6.3.2.1 Did you receive psychosocial support from healthcare professionals (CUMP, army health service, etc.)?

Oui –Non

Yes-No

##### A quel(s) moment(s) ?

*6.3.2.1.1 When? (More than one answer possible)*

Dans les 48 premières heures

Entre 2 et 7 jours après

Après la première semaine

Je ne sais plus

Within the first 48 hours

Between 2 and 7 days later

After the first week

I cannot remember

### Mairie du 11^e^

6.3.3 11^th^ district Town hall

#### Avez-vous bénéficié d’un soutien psychosocial des professionnels de santé (CUMP, service de santé des armées, etc) ?

6.3.3.1 Did you receive psychosocial support from healthcare professionals (CUMP, army health service, etc.)?

Oui-Non

Yes-No

##### A quel(s) moment(s) ?

6.3.3.1.1 *When? (More than one answer possible)*

Dans les 48 premières heures

Entre 2 et 7 jours après

Après la première semaine

Je ne sais plus

Within the first 48 hours

Between 2 and 7 days later

After the first week

I cannot remember

### Mairie de Saint-Denis ou centre municipal de santé rue du Cygne

6.3.4 Saint-Denis town hall or Municipal healthcare centre located in Rue du Cygne

#### Avez-vous bénéficié d’un soutien psychosocial des professionnels de santé (CUMP, service de santé des armées, etc) ?

6.3.4.1 Did you receive psychosocial support from healthcare professionals (CUMP, army health service, etc.)?

Oui-Non

Yes-No

##### A quel(s) moment(s) ?

6.3.4.1.1 *When? (More than one answer possible)*

Dans les 48 premières heures

Entre 2 et 7 jours après

Après la première semaine

Je ne sais plus

Within the first 48 hours

Between 2 and 7 days later

After the first week

I cannot remember

### Institut médico-légal

6.3.5 Institute of Forensic Medicine

#### Avez-vous bénéficié d’un soutien psychosocial des professionnels de santé (CUMP, service de santé des armées, etc) ?

6.3.5.1 Did you receive psychosocial support from healthcare professionals (CUMP, army health service, etc.)?

Oui-Non

Yes-No

##### A quel(s) moment(s) ?

6.3.5.1.1 *When? (More than one answer possible)*

Dans les 48 premières heures

Entre 2 et 7 jours après

Après la première semaine

Je ne sais plus

Within the first 48 hours

Between 2 and 7 days later

After the first week

I cannot remember

### Ecole Militaire

6.3.6 National Military school

#### Avez-vous bénéficié d’un soutien psychosocial des professionnels de santé (CUMP, service de santé des armées, etc) ?

6.3.6.1 Did you receive psychosocial support from healthcare professionals (CUMP, army health service, etc.)?

Oui-Non

Yes-No

##### A quel(s) moment(s) ?

6.3.6.1.1 *When? (More than one answer possible)*

Dans les 48 premières heures

Entre 2 et 7 jours après

Après la première semaine

Je ne sais plus

Within the first 48 hours

Between 2 and 7 days later

After the first week

I cannot remember

### Hôtel-Dieu

6.3.7 Hotel Dieu hospital

#### Avez-vous bénéficié d’un soutien psychosocial des professionnels de santé (CUMP, service de santé des armées, etc) ?

6.3.7.1 Did you receive psychosocial support from healthcare professionals (CUMP, army health service, etc.)?

Oui-Non

Yes-No

###### A quel(s) moment(s) ?

6.3.7.1.1.1 *When? (More than one answer possible)*

Dans les 48 premières heures

Entre 2 et 7 jours après

Après la première semaine

Je ne sais plus

Within the first 48 hours

Between 2 and 7 days later

After the first week

I cannot remember

6.3.8 AUTRE

6.3.8 Other

#### 6.3.8.1 Avez-vous bénéficié d’un soutien psychosocial des professionnels de santé (CUMP, service de santé des armées, etc) ?

6.3.8.1 Did you receive psychosocial support from healthcare professionals (CUMP, army health service, etc.)?

Oui-Non

Yes-No

##### 6.3.8.1.1 A quel(s) moment(s) ?

6.3.8.1.1 *When? (More than one answer possible)*

Dans les 48 premières heures

Entre 2 et 7 jours après

Après la première semaine

Je ne sais plus

Within the first 48 hours

Between 2 and 7 days later

After the first week

I cannot remember

## Depuis les évènements, en dehors des lieux cités précédemment êtes-vous allé voir ou avez-vous été reçu(e) par une (des) personne(s) d’un organisme, d’une association, d’un cabinet libéral pour vos difficultés psychologiques ?

**6.4 Since the events, aside from the places mentioned above, have you seen or been consulted by a person(s) from a public organization, an association or a private practice for your psychological problems?**

Oui – Non

Yes-No

### A quel organisme, association ou institution appartenai(en)t-il(s) ?

6.4.1 What public organisation, association or private practice was (were) the person(s) a part of?

Services d’urgence d’un hôpital

Consultation spécialisée en hôpital pour le psychotraumatisme

Consultation médico psychologique (CMP)

CUMP

Consultation spécialisée en secteur libéral

Association d’Aide aux victimes INAVEM (ex. Paris aide aux victimes, ADAVIP 92)

Association de victimes (AFVT –FENVAC)

Ose – Œuvre de secours aux enfants

Médecin traitant

Je ne sais pas

Autre

Hospital emergency services

Specialized hospital consultant for psychotrauma

Medical psychological Consultation (CMP)

CUMP

Specialized private consultant

An association in the “FRANCE VICTIMES” federation (e.g., ‘Paris aid to victims’, ADAVIP 92)

A victims’ association (e.g., AFVT -FENVAC)

The French medico-social children’s association Ose

A general practitioner

I don't know

Other

#### Si autre préciser

6.4.1.1 If other, specify which public organisation, association or private practice the person(s) was (were) part of?

## Depuis les événements, avez-vous été hospitalisé pour des difficultés psychologiques :

## 6.5 Since the events, have you been hospitalized for psychological problems?

Oui

Non

Yes-No

## Depuis les événements, avez-vous engagé des soins médico-psychologiques réguliers ?

## 6.6 Since the events, have you initiated regular psychological care?

Oui Non

Yes-No
