# Supplementary material for: Use of mental health supports by civilians exposed to the November 2015 terrorist attacks in Paris
Source: BMC Health Serv Res. 2020 Oct 20;20:959. doi: 10.1186/s12913-020-05785-3 (PMC7574168; doi:10.1186/s12913-020-05785-3)
Supplement: Supplementary file 3 — Additional file 3. Questions and criteria used to collect civilians’ self-reported information on use of mental-health supports (MHSu) for the ESPA_ 13_ November study. Additional file 3 contains two tables. Table A presents the questions used to collect self-reported information about the use of mental-health supports, table B presents the criteria used to distribute the answers to the different variable items according to the different types of MHSu. [file 12913_2020_5785_MOESM3_ESM.docx]

Additional file 3: Questions and criteria used to collect self-reported information on use of mental-health supports (MHSu), ESPA_13_November, phase 1, civilians

Table A: **Questions used to collect self-reported information about the use of mental-health supports, civilians, ESPA_13_November, N=454**

| **Questions used to collect information about declared mental health support use among civilian participants (N= 454)** | | |
| --- | --- | --- |
| **Qx** | **Title** | **Number** |
| **Q1** | **Do you have any memories of what you experienced between the immediate aftermath of the event and the moment when you returned to your living accommodation?**  *(Single choice : Yes, No)* | Yes=321 |
| Q2 | If *“Yes”* to Q1: Do you remember receiving any support or assistance?  *(Single choice : Yes, No)* | Yes=142 |
| Q3 | If *“Yes”* to Q2: Who provided this support or assistance ?  *(Multiple choice: Firefighters, SAMU, CUMP, Non- identified, Other Health Caregiver)* | 55 |
| Q4 | If answer to Q3: Can you tell us in which place(s)?  *(Multiple choice: At the site of the attack, In the street, Hôtel-Dieu, Other hospital, District town hall (Paris 10^th^ district, Paris 11^th^ district, St-Denis), Police office, Elsewhere)* | - |
| Q5 | If *“Elsewhere”* to Q4: Free-text clarification requested | 12 |
| **Q6** | **Afterr the events (or after returning to your living accommodation for people on or close to the scene at the time of the attacks/assault), did you go to any of the reception centres which were set up in the days following the attacks?**  *Multiple choice: Town Hall of the 10th district and adjacent school, Town Hall of the 11^th^, Saint-Denis town hall Institute of Forensic Medicine, Military School, Hôtel-Dieu, Other* | 227 |
| Q7 | If *“Other”* to Q6: Free-text clarification requested | 52 |
| Q8 | If answer to Q6: In these reception centre, did you receive psychosocial support from healthcare professionals (CUMP, army health service, etc.)? *(Single choice : Yes, No)* | Yes=108 |
| **Q9** | **Since the events, aside from the places mentioned above, have you seen or been consulted by a person(s) from a public organization, an association or a private practice for your psychological problems?**  *(Single choice : Yes, No)* | Yes=229 |
| Q10 | If *“Yes”* to Q9: What public organisation, association or private practice was (were) the person(s) a part of?  *(Multiple choice* *Hospital emergency services, Specialized hospital consultation service for psychotrauma, Medical psychological Centre (CMP), CUMP, Specialized private consultant, An association in the “FRANCE VICTIMES” federation (e.g., ‘Paris aid to victims’, ADAVIP 92), A victims’ association (e.g., AFVT -FENVAC), The French medico-social children’s association Ose, A general practitioner, I don't know, Other:* | - |
| Q11 | If *“Other”* to Q10:: Free-text clarification requested | 23 |
| **Q12** | **Since the events, have you been hospitalized for psychological problems?**  *(Single choice : Yes, Not)* | Yes=15 |
| **Q13** | **Were you physically injured during the attacks?**  *(Single choice : Yes, No)* | Yes=45 |
| Q14 | If *“Yes”* to Q13: Did the care which you initially received require emergency hospitalization that lasted for more than one week (Q14) Did the care which you initially received require emergency hospitalization that lasted for less than one week?(Q14bis))? Did the care you received require planned/deferred hospitalization (Q14ter)?  Oui-Non  *(Single choice : Yes, No)* | Yes=29 |
| Q15 | If *“Yes”* to Q14: Did you receive psychological support from a psychiatrist or psychologist during hospitalization?  *(Single choice : Yes, No)* | Yes=22 |
| **Q16** | **Since the events, have you initiated regular mental health care?**  *(Single choice : Yes, No)* | Yes=151 |

Table B: **Criteria used to collect self-reported information on use of mental-health supports (MHSu),** civilians, ESPA_ 13_ November, N=454

| **Criteria used to distribute the answers to the different questionnaire items according to the different types of MHSu** | | | |
| --- | --- | --- | --- |
| **Type of MHSU** | | **Questionnaire item number and selected possible answers** | **Numbers (%)** |
| **Outreach psychological support** | | | 159 (35) |
| Psychiatrist or psychologist in the street | | Q4 = *“At the place of the attack”,* *“In the street”* **OR**  Q5 = *various improvised places for delivering emergency psychological support were cited in the responses of this free-text question* | 29 |
| Occupational medicine | | Q 7 **OR** Q11 = cited business premises where ad hoc psychological support was provided | 7 |
| Psychologist of police | | Q4 = “*Police office”* **OR**  Q5 **OR** Q7 **OR** Q11= cited ad hoc psychological support at police office | 14 |
| MHSu in field-based reception centres | Hôtel-Dieu | Q4 = *“Hôtel-Dieu”* **OR** Q6 = *“Hôtel-Dieu”* **OR** Q11 = *“Hôtel-Dieu”*cited | 35 |
|  | Institute Forensic of Medicine | Q5 = cited *“Forensic Science Institute”* **OR** Q6 = *“Institute of Forensic Medicine”* **OR** Q11 = cited *“Forensic Science Institute”* | 8 |
|  | National Military school | Q5 = cited *“Military school”* **OR** Q6 = *”Military School”* | 15 |
|  | Town hall in affected district (Paris, St-Denis) ) | Q4 = *“City hall”* **OR** Q6 = *“Town Hall”* **OR** Q11 = specifying one of the 3 district city halls | 83 |
| Emergency psychosocial support unit (CUMP) | | Q3 = *”CUMP”* **OR** Q5 **OR** Q7 **OR** Q11 = cited psychological support by Emergency psychosocial support unit | 29 |
|  | |  |  |
| **Consultation with a specialist (psychiatrist or psychologist)** | | | 178 (39) |
| Specialized hospital consultant for psychotrauma | | Q10= *“Specialized hospital consultant for psychotrauma*  **OR** Q5 **OR** Q11 = specifying a visit to a psychiatric hospital service | 47 |
| Specialized private consultant | | Q10 = *“private practice”* **OR** Q5 **OR** Q11 = cited a visit to a private practice | 92 |
| Hospital emergency service | | Q4 =  *“Other hospital”* **OR** Q10 = *“Hospital emergency services ”* | 15 |
| Medical Psychological Centre (CMP) | | Q10 = *“CMP”* **OR** Q11 = cited a visit to a CMP | 36 |
| Hospitalised for psychological problems | | Q12 = *“Yes”* | 11 |
| Hospitalisation for physical injury | | Q13 = *“Yes”* **AND** Q14 = *“Yes”* **AND** Q15 = *“Yes”* | 22 |
| **Contact with a member of an association** | | | **71 (16)** |
| Victims’ support association (FRANCE VICTIMES federation) | | Q10 = *“victim support association”* **OR**  Q11 = cited contact with a victim support association, | 44 |
| Victims’ association | | Q10 = *“victims' association“* **OR**  Q11 = cited contact with a victims' association or victim support association | 35 |
| **Medical visit to a GP's office (GP)** | | Q10 = *“general practitioner”* **OR** Q11 = cited visiting a general practitioner | **79 (17)** |
| **Initiated regular mental health treatment** | | Q16 = *“Yes”* | **151 (33)** |
